# Preventive Attenuation of MPTP-Induced Parkinson’s Disease-like Abnormalities by *Lactiplantibacillus plantarum* SHOU LAC-1 in Mice

**DOI:** 10.3390/foods15173116

**Published:** 2026-09-02

**Authors:** Wenrui Zhao, Yaqin He, Houxiang Ma, Dongpin Tu, Jun Chen, Quancai Sun, Ye Peng, Zhiyao An, Pengcheng Pan, Wenhui Wu, Xichang Wang, Fengwei Tian, Wanqiang Wu, Jianxin Zhao

**Affiliations:** 1 College of Food Science and Technology, Shanghai Ocean University, Shanghai 201306, China; riozhao@foxmail.com (W.Z.); heyaqin0807@163.com (Y.H.); 17658155875@163.com (H.M.); 15240816242@163.com (D.T.); 15587569635@163.com (Z.A.); 19826221108@163.com (P.P.); xcwang@shou.edu.cn (X.W.); 2 College of Life and Health, West Anhui University, Lu’an 237000, China; junchen@wxc.edu.cn; 3 Faculty of Medicine, Macau University of Science and Technology, Taipa, Macao SAR, China; qcsun@must.edu.mo (Q.S.); pengye@must.edu.mo (Y.P.); 4 Department of Marine Pharmacology, College of Food Science and Technology, Shanghai Ocean University, Shanghai 201306, China; whwu@shou.edu.cn; 5 Putuo Sub-Center of International Joint Research Center for Marine Biological Sciences, Zhoushan 316104, China; 6 Marine Biomedical Science and Technology Innovation Platform of Lin-Gang Special Area, Shanghai 201306, China; 7 Shanghai Engineering Research Center of Aquatic-Product Processing and Preservation, Shanghai 201306, China; 8 State Key Laboratory of Food Science and Resources, Jiangnan University, Wuxi 214122, China; fwtian@jiangnan.edu.cn; 9 School of Food Science and Technology, Jiangnan University, Wuxi 214122, China; 10 National Engineering Research Center for Functional Food, Jiangnan University, Wuxi 214122, China; 11 International Research Center for Food and Health, College of Food Science and Technology, Shanghai Ocean University, Shanghai 201306, China

**Keywords:** Parkinson’s disease, *Lactiplantibacillus plantarum* SHOU LAC-1, microbiota–gut–brain axis, gut microbiota, neuroinflammation, serum metabolomics

## Abstract

Dysregulation of the microbiota–gut–brain axis has been implicated in Parkinson’s disease (PD), and modulation of the intestinal microbiota may offer a complementary nutritional approach. This study evaluated the preventive attenuation of MPTP-induced PD-like abnormalities by *Lactiplantibacillus plantarum* SHOU LAC-1 and examined associated microbiota–gut–brain axis-related changes. Forty male C57BL/6J mice were allocated to Control, MPTP, L-DOPA, and SHOU LAC-1 groups (*n* = 10 per group). SHOU LAC-1 was orally administered at 1 × 10^9^ CFU per mouse per day for 42 days, with MPTP administered during the final 14 days; L-DOPA was administered during the MPTP-treatment period as a positive control. Motor performance and defecatory parameters, nigrostriatal TH and α-SYN immunoreactivity, neurotrophic-support-related gene expression, inflammatory markers, glial-cell-related gene expression, Nrf2-related antioxidant gene expression, colonic histopathology and tight-junction-related gene expression, gut microbiota composition, and serum metabolic profiles were assessed. Compared with MPTP-treated mice, SHOU LAC-1-treated mice showed better motor and defecatory performance, partial preservation of nigrostriatal TH immunoreactivity, reduced α-SYN immunoreactivity, increased neurotrophic-support-related gene expression, lower central and colonic inflammatory markers and glial-cell-related gene expression, increased Nrf2/HO-1/NQO1-related antioxidant gene expression, and comparatively better-preserved colonic morphology with higher tight-junction-related gene expression. 16S rRNA sequencing and untargeted serum metabolomics further showed that SHOU LAC-1 administration was accompanied by changes in gut microbial diversity and composition and serum metabolic profiles. Overall, under this preventive experimental design, SHOU LAC-1 administration was associated with attenuation of multiple MPTP-induced PD-like abnormalities, accompanied by changes in gut microbial and serum metabolic profiles. These preclinical findings are predominantly associative; causal relationships among microbial, metabolic, intestinal, and neurological changes remain to be established, and the translational relevance of SHOU LAC-1 requires further validation.

## 1. Introduction

Parkinson’s disease (PD) is among the most prevalent neurodegenerative disorders worldwide, and its incidence and prevalence increase markedly with age. As population aging continues to accelerate globally, the public health burden associated with PD is projected to increase substantially in the coming decades. Modeling analyses based on the Global Burden of Disease 2021 dataset have estimated that approximately 25.2 million individuals may be living with PD worldwide by 2050 [1,2]. The principal neuropathological features of PD include the progressive degeneration of dopaminergic neurons in the substantia nigra pars compacta, impairment of nigrostriatal dopaminergic projections, and abnormal aggregation of α-synuclein (α-SYN) [3,4,5]. These pathological alterations are closely associated with the classical motor manifestations of PD, including bradykinesia, resting tremor, muscular rigidity, postural instability, and gait disturbance. At present, dopamine replacement therapy, particularly levodopa (L-DOPA), remains the main pharmacological approach for alleviating motor symptoms in patients with PD. However, currently available treatments are largely symptomatic and have not been demonstrated to prevent or reverse the underlying neurodegenerative process. Moreover, long-term dopaminergic therapy may be accompanied by motor fluctuations, dyskinesia, and other treatment-related complications [3,4]. Therefore, there remains considerable interest in safe and sustainable adjunctive approaches that may address PD-related dysfunction beyond symptomatic dopaminergic replacement.

Beyond motor impairment, PD is characterized by a broad spectrum of non-motor symptoms, among which gastrointestinal dysfunction is particularly common. Constipation, delayed intestinal transit, and defecatory abnormalities may occur years before the emergence of classical motor symptoms and are increasingly recognized as clinically relevant features of the prodromal phase of PD [3,6]. These observations suggest that the initiation and progression of PD may not be restricted to the central nervous system but may also involve peripheral organs, particularly the gastrointestinal tract. Bidirectional communication between the gut and the central nervous system is mediated through neural, immune, endocrine, and metabolic pathways, which together constitute the microbiota–gut–brain axis (MGBA) [6,7,8,9]. Within this network, the gut microbiota is involved not only in maintaining intestinal ecological homeostasis, but also in processes related to epithelial barrier integrity, immune and inflammatory responses, neuroactive molecule production, and host metabolic status. Accordingly, alterations in the intestinal microbial ecosystem have been associated with central nervous system homeostasis and PD-related pathological processes [7,8,9].

Increasing evidence from clinical cohorts, metagenomic analyses, and experimental models has associated gut microbial dysbiosis with PD-related abnormalities. Compared with healthy individuals, patients with PD have frequently been reported to exhibit altered gut microbial composition and microbial functional profiles, including reduced abundance of several taxa previously associated with short-chain fatty acid (SCFA) production and increased abundance of certain inflammation-associated or potentially pathogenic taxa [10,11,12,13,14]. Metagenomic studies have further suggested that PD-associated microbial alterations may be linked to inflammatory regulation, neuroactive molecule metabolism, lipid and energy metabolism, and the depletion of potentially neuroprotective microbial metabolites [11,14]. In particular, reductions in taxa previously associated with SCFA production, together with lower SCFA levels, have been associated with gastrointestinal symptoms, impaired intestinal barrier function, and disease progression in PD [12,15,16]. However, such associations do not establish a causal contribution of particular microbial taxa or SCFA-related changes to PD. Nevertheless, the role of gut microbial dysbiosis in PD remains incompletely defined. It is still debated whether microbial alterations represent contributors to disease progression, secondary consequences of disease development, or integrated outcomes shaped by diet, medication, disease stage, and host physiology [6,13,14]. Further mechanistic studies are therefore needed to clarify how gut microbial changes may participate in PD-related gut–brain interactions.

Intestinal alterations, inflammatory and oxidative-stress-related responses, gut microbial composition, and host metabolism have all been implicated in PD-related gut–brain interactions [7,13,14,17,18,19,20]. Gut microbiota-derived metabolites may also be associated with inflammatory, oxidative, metabolic, and neuroimmune processes relevant to the MGBA. Nevertheless, the relationships among these central and peripheral alterations remain complex, and their causal organization has not been fully established. Accordingly, integrated assessment of intestinal, microbial, inflammatory, antioxidant-related, metabolic, and neurological changes may provide a more comprehensive understanding of PD-related gut–brain interactions without presupposing a causal pathway among these biological domains.

Probiotic intervention has been increasingly investigated as a nutritional strategy targeting gut-related abnormalities associated with PD. Clinical studies and systematic reviews have indicated that probiotic or synbiotic supplementation may improve constipation in patients with PD to some extent and may also be associated with changes in motor performance, inflammatory status, and gut–brain axis-related outcomes [21,22,23]. However, the biological effects of probiotics are generally strain-specific and may be influenced by strain origin, host condition, disease stage, dosage, and intervention duration. Therefore, findings obtained from one probiotic strain should not be directly extrapolated to another. These strain-specific differences highlight the need to evaluate individual candidate strains within appropriate experimental models.

*Lactiplantibacillus plantarum* is widely present in fermented foods and gastrointestinal environments and has attracted attention because of its environmental adaptability and functional diversity [24]. Previous animal studies have suggested that certain *L. plantarum* strains may alleviate motor deficits and neuropathological alterations in PD-like models, with these effects being accompanied by changes in gut microbiota composition, microbial metabolite-related pathways, neuroinflammation, and oxidative stress [25,26,27,28,29]. However, it remains unknown whether *L. plantarum* SHOU LAC-1 can preventively attenuate MPTP-induced PD-like abnormalities. Moreover, whether SHOU LAC-1-associated phenotypic changes occur in parallel with coordinated alterations in gut microbial composition, intestinal indicators, inflammatory and antioxidant-related responses, and systemic metabolic profiles has not yet been systematically investigated. We hypothesized that preventive administration of SHOU LAC-1 would attenuate selected MPTP-induced PD-like abnormalities and that these phenotypic changes would be accompanied by coordinated central and peripheral alterations relevant to the MGBA.

A distinctive aspect of the present study is the integrated evaluation of central and peripheral outcomes within an MGBA framework. Behavioral and neuropathological assessments were integrated with analyses of gastrointestinal function, inflammatory and antioxidant-related responses, gut microbial composition, and serum metabolic profiles. This multidimensional strategy was designed to characterize coordinated changes associated with SHOU LAC-1 administration rather than to establish a causal microbiota–metabolite–gut–brain pathway. L-DOPA was included solely as a positive control to demonstrate the responsiveness of the experimental model, and the study was not designed to compare the relative efficacy of SHOU LAC-1 and L-DOPA. Because SHOU LAC-1 administration began before MPTP exposure, the experimental design represents a preventive/protective intervention paradigm rather than treatment of established PD-like injury. Accordingly, the present study aimed to evaluate whether SHOU LAC-1 attenuates MPTP-induced PD-like abnormalities and whether these changes are accompanied by coordinated central and peripheral alterations relevant to the MGBA.

## 2. Materials and Methods

### 2.1. Animals and Experimental Design

Male C57BL/6J mice aged 6–8 weeks and weighing 18–20 g were obtained from Spefo Biotechnology Co., Ltd. (Suzhou, Jiangsu, China). All animals were maintained under specific pathogen-free conditions in a controlled environment with a 12 h light/dark cycle, an ambient temperature of 22 ± 2 °C, and a relative humidity of 55 ± 10%. Standard rodent chow and drinking water were provided ad libitum throughout the experimental period. The Animal Ethics Committee of Shanghai Ocean University approved all animal experiments (protocol number: SHOU-DW-2025-184).

**Figure 1:**
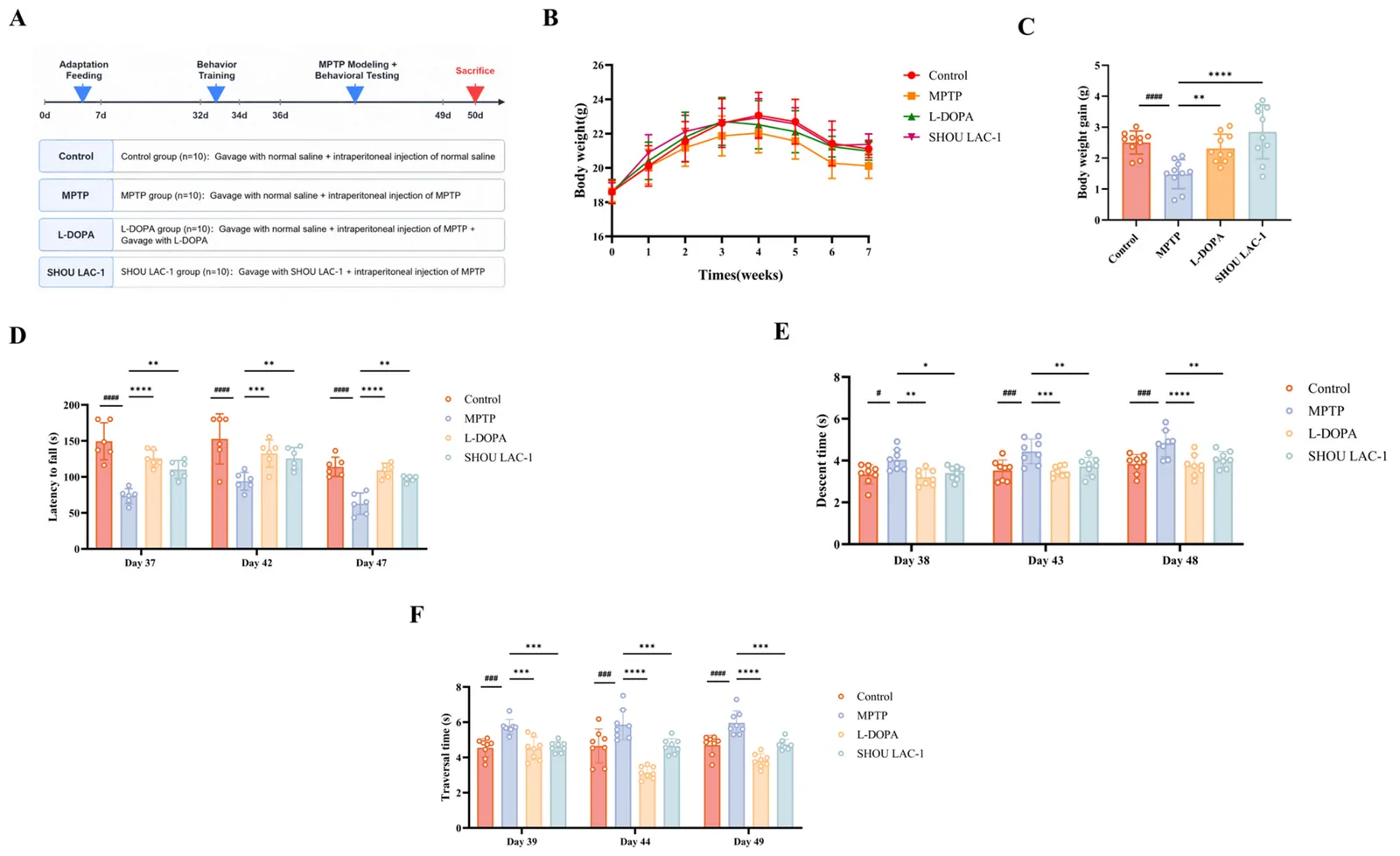
*Lactiplantibacillus plantarum* SHOU LAC-1 was associated with attenuation of MPTP-induced reduction in body-weight gain and motor dysfunction in mice. (**A**) Timeline of the animal experiment and treatment schedule. (**B**) Changes in body weight during the experimental period. (**C**) Body-weight gain during the experiment. (**D**) Rotarod latency to fall assessed on Days 37, 42, and 47. (**E**) Pole descent time assessed on Days 38, 43, and 48. (**F**) Balance beam traversal time assessed on Days 39, 44, and 49. Data are presented as the mean ± SD. For body-weight measurements and body-weight gain, *n* = 10 mice per group; for the rotarod test, *n* = 6 mice per group; and for the pole and balance beam tests, *n* = 8 mice per group. At each behavioral testing session, each mouse completed three within-session trials, and the mean of the three trials was used as the session-level outcome for that animal. Individual within-session trials were not treated as independent observations. Longitudinal body-weight data and repeated behavioral-session data were analyzed using two-way repeated-measures ANOVA. # *p* < 0.05, ### *p* < 0.001, and #### *p* < 0.0001 versus the Control group; * *p* < 0.05, ** *p* < 0.01, *** *p* < 0.001, and **** *p* < 0.0001 versus the MPTP group.

The overall experimental schedule is shown in Figure 1A. After a 7-day acclimatization period, 40 male C57BL/6J mice were allocated to four experimental groups (*n* = 10 per group at study initiation): Control, MPTP, L-DOPA, and SHOU LAC-1. Group allocation was performed using a body-weight-stratified procedure, whereby mice across the baseline body-weight range were distributed among the four groups to achieve comparable initial body-weight profiles. No formal a priori sample-size or statistical power calculation was performed. Formal investigator blinding was not prospectively documented; therefore, a strictly blinded experimental design is not claimed. Male mice were used to maintain consistency with commonly used MPTP-induced PD-like mouse protocols [30,31,32,33].

From day 8 to day 49, mice in the Control and MPTP groups were administered 200 μL of sterile saline once daily by oral gavage. Mice in the SHOU LAC-1 group were administered *Lactiplantibacillus plantarum* SHOU LAC-1 suspension at a dose of 1 × 10^9^ CFU in 200 μL of sterile saline per mouse per day. The MPTP-induced mouse model has been widely used to investigate PD-like dopaminergic abnormalities and motor dysfunction [30,31,32]. From day 36 to day 49, mice in all groups except the Control group received daily intraperitoneal injections of MPTP hydrochloride at 20 mg/kg [33], whereas mice in the Control group received an equivalent volume of sterile saline.

MPTP hydrochloride (HY-15608), L-DOPA (HY-N0304), and benserazide hydrochloride (HY-B0404A) were purchased from MedChemExpress (Monmouth Junction, NJ, USA). Mice in the L-DOPA group were administered 200 μL of sterile saline once daily by oral gavage from day 8 to day 35. During the MPTP modeling period, mice in this group were administered L-DOPA at 100 mg/kg in combination with benserazide at 25 mg/kg by oral gavage. Body weight was recorded weekly, and fecal samples were collected according to the experimental schedule. Behavioral tests were performed during the modeling period on separate days in a fixed order. Behavioral assessments were performed during the modeling period as described below.

On Day 50, mice were anesthetized by intraperitoneal injection of Delivector™ Avertin (DW3101; Dowobio, Shanghai, China; tribromoethanol) at 20 μL/g body weight. Blood was collected under anesthesia, after which the mice were euthanized by cervical dislocation and tissues were collected for subsequent analyses. No animals were excluded from the experimental cohort, and no animal deaths or serious adverse events occurred during the study.

### 2.2. Preparation of Lactiplantibacillus plantarum SHOU LAC-1 Suspension

*Lactiplantibacillus plantarum* SHOU LAC-1 was isolated from a pickled Chinese cabbage sample collected from Chuzhou, Anhui Province, China. The strain was taxonomically identified as *L. plantarum* by 16S rRNA gene sequencing followed by sequence comparison against the NCBI database and was deposited in the China General Microbiological Culture Collection Center under accession number CGMCC No. 38417.

For activation, the glycerol-preserved strain was streaked onto de Man, Rogosa, and Sharpe (MRS) agar and cultured anaerobically at 37 °C for 48 h to obtain single colonies. MRS agar and broth were purchased from Beijing Land Bridge Technology Co., Ltd. (Beijing, China). A single colony was inoculated into MRS broth and cultured anaerobically at 37 °C overnight. The activated culture was then transferred into fresh MRS broth at an inoculation ratio of 1% (*v*/*v*) and cultured under the same conditions for 24 h.

Viable bacterial counts were determined by serial dilution and spread plating on MRS agar, and the resulting CFU counts were used to standardize the bacterial concentration. Bacterial cells were harvested by centrifugation at 8000× *g* for 10 min at 4 °C and washed twice with sterile 0.9% saline. Before oral gavage, the bacterial cells were resuspended in sterile saline to a final concentration of 5 × 10^9^ CFU/mL. Each mouse received 200 μL per day, corresponding to approximately 1 × 10^9^ CFU of SHOU LAC-1 per mouse per day.

### 2.3. Behavioral Tests and Gastrointestinal Function Assessment

Motor function was evaluated using the rotarod test, pole test, and balance beam test. With the first day of acclimatization defined as experimental Day 1, the rotarod test was performed on Days 37, 42, and 47; the pole test on Days 38, 43, and 48; and the balance beam test on Days 39, 44, and 49. Behavioral tests were conducted on separate days under standardized testing conditions to reduce potential interference caused by fatigue and stress.

At each assessment session, each mouse completed three trials, and the mean of the three trials was used as the session-level value. Thus, the three time points for each behavioral test represent three repeated assessment sessions conducted on separate days rather than three independent trials within a single session. Session-level values repeatedly obtained from the same animals were treated as repeated measurements across time in the statistical analysis. Formal investigator blinding to group allocation was not implemented during behavioral testing; however, all tests were conducted according to predefined standardized procedures using objective time-based endpoints. These behavioral paradigms are commonly used to assess neurotoxin-induced motor abnormalities in PD-related animal models [34].

For the rotarod test, mice were trained before modeling at 10 r/min and 20 r/min for 180 s at each speed. During formal testing, the rotation speed was set at 30 r/min, and the latency to fall was recorded. At each assessment session, each mouse completed three trials with an interval of 30 min between trials, and the mean of the three trials was used as the session-level outcome.

For the pole test, each mouse was placed head-upward on the top of a rough-surfaced vertical pole measuring 50 cm in length and 1 cm in diameter. The time required for the hindlimbs to reach the base of the pole was recorded, with a maximum cutoff time of 60 s. At each assessment session, each mouse completed three trials, and the mean of the three trials was used as the session-level outcome.

For the balance beam test, each mouse was placed on a narrow beam measuring 80 cm in length, with a dark box positioned at the endpoint. The time required to traverse the beam was recorded. At each assessment session, each mouse completed three trials, and the mean of the three trials was used as the session-level outcome.

Gastrointestinal function was assessed before terminal sample collection. Each mouse was individually placed in a clean and sterilized container for 30 min, during which freshly excreted fecal pellets were collected. The number of fecal pellets was recorded, and the wet weight was measured immediately. A portion of each fecal sample was stored at −80 °C for subsequent analyses, whereas the remaining feces were dried at 90 °C for 24 h to determine the dry weight. Fecal water content was calculated as follows: (wet weight − dry weight)/wet weight × 100%.

### 2.4. Sample Collection and Tissue Preparation

After completion of the behavioral assessments, mice were fasted overnight with free access to water. On Day 50, mice were anesthetized by intraperitoneal administration of Avertin (tribromoethanol; 20 μL/g body weight), and blood was collected from the retro-orbital venous plexus. Blood samples were allowed to clot at room temperature for 30 min. The centrifuge was precooled to 4 °C before use. Serum was separated by centrifugation at approximately 625× *g* (3000 r/min) for 15 min at 4 °C using a TG20.5 centrifuge equipped with a 12 × 1.5/2.2 mL fixed-angle rotor (Shanghai Lu Xiangyi Centrifuge Instrument Co., Ltd., Shanghai, China), aliquoted, and stored at −80 °C until metabolomic analysis.

Following blood collection, mice were euthanized by cervical dislocation under anesthesia. Brain and colon tissues were rapidly collected and processed on ice. The midbrain was collected for inflammatory cytokine assays, whereas the striatum was collected for gene-expression analyses of inflammatory-, glial-cell-related, dopaminergic, neurotrophic, and Nrf2-related antioxidant markers. Brain tissues designated for immunohistochemical analysis were fixed in 4% paraformaldehyde.

An approximately 1–2 cm long segment of the distal colon was collected, gently rinsed with pre-cooled phosphate-buffered saline to remove intestinal contents, and blotted dry. A portion of the colon tissue was fixed in 4% paraformaldehyde for histological analysis, whereas the remaining tissue was snap-frozen in liquid nitrogen and stored at −80 °C for subsequent molecular and biochemical analyses.

The animal experiment was initiated with 10 mice per group. All animals were included in body-weight monitoring and fecal measurements and were scheduled for behavioral assessment. For the individual behavioral assays, complete valid assay-specific measurements were available for six mice per group in the rotarod test and eight mice per group in the pole and balance beam tests; therefore, these assay-specific sample sizes were used for the corresponding analyses. These assay-specific sample sizes did not represent exclusion from the experimental cohort.

Four individual mice per group were used for immunohistochemical analyses and colon H&E staining. Four biological samples per group were used for RT-qPCR analyses, whereas three biological samples per group were used for ELISA analyses. Ten fecal samples per group were subjected to 16S rRNA gene sequencing. The final exploratory microbiota analyses were conducted using the six biological samples per group included in the original bioinformatics analysis workflow. Six serum samples per group were subjected to untargeted metabolomic profiling. The final exploratory metabolomic analyses were conducted using the four biological samples per group included in the original metabolomic data-analysis workflow. Exact assay-specific biological sample sizes are also specified in the corresponding method subsections and figure legends.

### 2.5. Hematoxylin and Eosin Staining

Colon tissue samples were fixed in 4% paraformaldehyde for 24 h, dehydrated through a graded ethanol series, embedded in paraffin, and sectioned at a thickness of 5 μm. The sections were stained with hematoxylin and eosin, followed by dehydration, clearing, and mounting. Four biological samples per group were examined (*n* = 4 per group). Representative colonic images were examined at 200× and 400× magnification. Colonic morphological alterations were evaluated qualitatively based on tissue architecture and mucosal morphology. Because no predefined semiquantitative histopathological scoring system was applied, the histological observations were treated as descriptive rather than quantitative histopathological outcomes. Formal investigator blinding during histological evaluation was not prospectively documented; therefore, a strictly blinded histological assessment is not claimed.

### 2.6. Immunohistochemistry

Paraffin-embedded brain tissues were sectioned at a thickness of 5 μm. The sections were deparaffinized and rehydrated, and heat-induced antigen retrieval was performed in citrate antigen retrieval buffer (pH 6.0) at 100 °C for 15 min. After natural cooling, the sections were washed three times with phosphate-buffered saline and incubated with 3% hydrogen peroxide at room temperature in the dark for 25 min to block endogenous peroxidase activity. The sections were then blocked with 3% bovine serum albumin at room temperature for 30 min and incubated with primary antibodies overnight at 4 °C.

The primary antibodies included anti-tyrosine hydroxylase antibody (TH; GB11181; rabbit IgG; 1:1000; Servicebio, Wuhan, China) and anti-α-synuclein antibody (α-SYN; GB112945; rabbit IgG; 1:1500; Servicebio, Wuhan, China). After washing, the sections were incubated with an S-vision immunohistochemical polymer secondary antibody against rabbit IgG (G1302; ready to use; Servicebio, Wuhan, China) at room temperature for approximately 50 min. Immunoreactivity was visualized using 3,3′-diaminobenzidine, followed by hematoxylin counterstaining, dehydration through a graded ethanol series, clearing in xylene, and mounting.

For each immunohistochemical marker, four biological samples from four individual mice per group were analyzed (*n* = 4 per group). For each immunohistochemical marker, one anatomically comparable section from each animal was used for quantitative analysis. Sections were selected according to consistent anatomical landmarks across all experimental groups. The stained sections were digitized using a slide scanner to obtain whole-slide images. Corresponding anatomical regions of interest were identified on the digitized sections, and representative regions were digitally enlarged and exported for figure preparation. Multiple regions, screenshots, or measurements obtained from the same animal were not treated as independent biological replicates.

Quantitative image analysis was performed using ImageJ2 [35], with consistent region-of-interest selection criteria and image-analysis procedures across all experimental groups. TH and α-SYN immunoreactivity was quantified as the percentage of positively stained area within the corresponding anatomical region of interest. Each individual animal represented one biological replicate for statistical analysis. Formal investigator blinding during digital image selection and quantitative analysis was not prospectively documented; therefore, a strictly blinded image analysis is not claimed.

### 2.7. Reverse Transcription-Quantitative Polymerase Chain Reaction

Total RNA was extracted from striatal and colonic tissues using a total RNA extraction kit (AG21017; Accurate Biotechnology Co., Ltd., Hunan, China) according to the manufacturer’s instructions. RNA concentration and purity were assessed using a NanoDrop spectrophotometer, and RNA integrity was evaluated by agarose gel electrophoresis. The extracted RNA was reverse-transcribed into complementary DNA using Hifair^®^ III 1st Strand cDNA Synthesis SuperMix for qPCR with gDNA digester plus (11141ES60; Yeasen Biotechnology Co., Ltd., Shanghai, China). Quantitative polymerase chain reaction was performed using Hieff UNICON^®^ Universal Blue qPCR SYBR Green Master Mix (11184ES08; Yeasen Biotechnology Co., Ltd., Shanghai, China) on a SLAN automated medical PCR detection system. Four biological samples from four individual mice per group were analyzed for each RT-qPCR outcome (*n* = 4 per group). Each biological sample was analyzed in three technical replicates in a total reaction volume of 20 μL, and the mean Ct value of the technical replicates was used for subsequent analysis. The cycling conditions were 95 °C for 2 min, followed by 40 cycles of 95 °C for 10 s and 60 °C for 30 s, followed by melting-curve analysis. No-template and no-reverse-transcription controls were included.

In the striatum, the mRNA expression levels of inflammatory-related genes (*Tnf*, *Il1b*, and *Il6*), glial-cell-associated genes (*Gfap* and *Aif1*), dopaminergic- and neurotrophic-related genes (*Th*, *Bdnf*, and *Gdnf*), and Nrf2-related antioxidant-response genes (*Nfe2l2*, *Nqo1*, and *Hmox1*) were determined. In the colon, the mRNA expression levels of inflammatory-related genes (*Tnf*, *Il1b*, and *Il6*) and tight-junction-related genes (*Tjp1*, *Ocln*, and *Cldn1*) were measured.

Cycle threshold values were obtained, and relative gene expression was calculated using the 2^−ΔΔCt^ method [36], with *Gapdh* used as the internal reference gene. Primer amplification efficiencies were not independently determined, and the stability of *Gapdh* expression across experimental groups was not formally evaluated in the present study. Primer sequences are provided in the Supplementary Materials.

### 2.8. Enzyme-Linked Immunosorbent Assay

The protein levels of the inflammatory cytokines TNF-α, IL-1β, and IL-6 were measured in mouse midbrain and colon tissues using commercial mouse enzyme-linked immunosorbent assay (ELISA) kits purchased from Shanghai Jianglai Biotechnology Co., Ltd. (Shanghai, China), according to the manufacturer’s instructions. The catalog numbers were JL10484 for TNF-α, JL18442 for IL-1β, and JL20268 for IL-6.

Tissues were rinsed with ice-cold phosphate-buffered saline (PBS; 0.01 M, pH 7.4), weighed, minced, and homogenized on ice in ice-cold PBS at a tissue-to-buffer ratio of 1:9 (*w*/*v*). The homogenates were centrifuged at 5000× *g* for 5–10 min, and the resulting supernatants were collected for ELISA analysis. Three biological samples from three individual mice per group were analyzed for each tissue and cytokine assay (*n* = 3 per group). Each biological sample was analyzed in three technical replicate wells, and the mean value of the three replicate wells was used as the value for that individual animal.

Samples and standards were assayed according to the manufacturer’s protocol. Cytokine concentrations were calculated from the corresponding standard curves. The assay sensitivities were 7.26 pg/mL for TNF-α, 1.98 pg/mL for IL-1β, and 2.63 pg/mL for IL-6. According to the manufacturer, the intra- and inter-assay coefficients of variation were <10%. Absorbance was measured at 450 nm using a Bio-Tek ELX-800 microplate reader (BioTek Instruments, Inc., Winooski, VT, USA). Cytokine concentrations were normalized to the original tissue weight and expressed as pg/mg tissue.

### 2.9. Gut Microbiota and Bioinformatics Analysis

Total microbial DNA was extracted from fecal samples using a fecal genomic DNA extraction kit (DP328; TIANGEN BIOTECH (BEIJING) CO., LTD., Beijing, China). No extraction blank was included during the DNA extraction procedure. The V4 region of the bacterial 16S rRNA gene was amplified using the 515F/806R primer pair, with a negative PCR control included. PCR products were purified and used for library construction. Qualified libraries were quantified and subjected to PE250 paired-end sequencing on an Illumina NovaSeq 6000 platform by Novogene Co., Ltd. (Beijing, China).

Raw paired-end reads were quality filtered, merged, and screened for chimeras. Amplicon sequence variants (ASVs) were generated using the DADA2 module implemented in QIIME2 version 2022.2 (QIIME2-202202), and taxonomic annotation was performed against SILVA release 138.1 [37,38,39]. Sequence abundance was rarefied to 80,907 reads per sample before diversity analyses. Alpha diversity was evaluated using the Chao1, Observed features, and Shannon indices. Differences in alpha-diversity indices among groups were assessed using the Kruskal–Wallis rank-sum test, followed by the Conover–Iman post hoc test for pairwise comparisons.

Beta diversity was evaluated using weighted and unweighted UniFrac distances and visualized by principal coordinate analysis (PCoA); principal component analysis (PCA) was additionally used for ordination visualization. Differences in microbial community composition among groups were assessed using PERMANOVA (Adonis) based on Bray–Curtis distances with 999 permutations.

Ten fecal samples per group were subjected to 16S rRNA gene sequencing. The final exploratory microbiota analyses were conducted using six biological samples per group (*n* = 6 per group), corresponding to the sample subset used in the original bioinformatics analysis workflow. Six biological samples per group were included in the LEfSe analysis (*n* = 6 per group). Differentially abundant taxa from the phylum to genus levels were identified using linear discriminant analysis effect size (LEfSe), with a Kruskal–Wallis threshold of *p* < 0.05 and an LDA score threshold of 2.0. A one-against-one comparison strategy was applied, and unclassified taxa were retained. LEfSe findings were interpreted as exploratory taxonomic associations rather than direct evidence of microbial function.

### 2.10. Untargeted Serum Metabolomics

Untargeted metabolomic profiling of mouse serum samples was performed by Shanghai Biotree Biotech Co., Ltd. (Shanghai, China) using an ultra-high-performance liquid chromatography–tandem mass spectrometry platform. Serum samples were thawed on ice, and 50 μL of each sample was mixed with 200 μL of methanol/acetonitrile (1:1, *v*/*v*) extraction solution containing isotope-labeled internal standards. Samples were shaken at 750 rpm for 5 min, allowed to stand for 5 min, and filtered through a protein-precipitation plate. Quality control (QC) samples were prepared by pooling equal aliquots of the processed samples. Experimental samples were analyzed in a randomized injection order, and one QC sample was injected after every nine experimental samples.

Chromatographic separation was performed using a Vanquish UHPLC system (Thermo Fisher Scientific, Germering, Germany) coupled to an Orbitrap Exploris 120 high-resolution mass spectrometer with a Waters ACQUITY UPLC BEH Amide column (2.1 × 50 mm, 1.7 μm). Mobile phase A consisted of 25 mmol/L ammonium acetate and 25 mmol/L ammonium hydroxide in water (pH 9.75), and mobile phase B was acetonitrile. The autosampler temperature was maintained at 4 °C, the column temperature was 30 °C, the injection volume was 2 μL, and the flow rate was 0.50 mL/min.

The mass-spectrometric settings included a sheath gas flow rate of 50 Arb, an auxiliary gas flow rate of 15 Arb, a capillary temperature of 320 °C, a full-MS resolution of 60,000, an MS/MS resolution of 15,000, stepped normalized collision energies of 20/30/40, and spray voltages of 3.8 kV and −3.4 kV in positive and negative ionization modes, respectively. The full-MS scan range was m/z 70–1200. Data acquisition was controlled using Xcalibur version 4.4.

Raw data were converted to mzXML format using ProteoWizard V3.0.24054 and processed using an R/XCMS-based workflow for feature detection, extraction, alignment, and integration. Metabolite annotation was performed using BiotreeDB V3.0 based on MS1, MS2, and retention-time information, and identification confidence was annotated according to Metabolomics Standards Initiative levels. Data preprocessing included RSD-based feature filtering, missing-value filtering, imputation of missing values using one-half of the minimum value, and total ion current normalization.

Six biological serum samples per group were subjected to untargeted metabolomic profiling. The final exploratory metabolomic analyses were based on four biological samples per group (*n* = 4 per group), corresponding to the sample subset used in the original metabolomic data-analysis workflow. The processed data were imported into SIMCA 18.0.1 for multivariate analysis. PCA was used for unsupervised visualization, whereas OPLS-DA was used as an exploratory supervised model. OPLS-DA models were evaluated using seven-fold cross-validation and 200 permutation tests, with R^2^ and Q^2^ used to assess model fit and predictive performance. Differential metabolic features were initially screened using VIP > 1.0 and nominal *p* < 0.05. To account for multiple testing, *p*-values were further adjusted using the Benjamini–Hochberg false-discovery-rate procedure; features with *q* < 0.05 were considered FDR-supported, whereas features not surviving FDR correction were interpreted as exploratory. Pathway annotation and enrichment analysis were performed using KEGG and MetaboAnalyst [40,41].

### 2.11. Statistical Analysis

Statistical analyses were performed using GraphPad Prism 10.1 unless otherwise specified. Data are presented as the mean ± standard deviation unless otherwise stated.

Longitudinal body-weight data and repeated behavioral-session data were analyzed using two-way repeated-measures ANOVA, with group as the between-subject factor and time or testing session as the within-subject factor, including the group × time/session interaction. For behavioral assessments, the mean of the three within-session trials was used as the session-level outcome for each animal, and individual within-session trials were not treated as independent observations. Where applicable, Dunnett’s multiple-comparisons test was used for comparisons between treatment groups and the MPTP group.

For conventional endpoint analyses, normality of model residuals was assessed using the Shapiro–Wilk test, and homogeneity of variance among groups was evaluated using the Brown–Forsythe test. Parametric analyses were used when the relevant assumptions were reasonably satisfied. For HO-1, marginal heterogeneity of variance was detected by the Brown–Forsythe test (*p* = 0.0496); therefore, Welch’s ANOVA was additionally performed as a sensitivity analysis and yielded the same overall statistical conclusion.

For endpoint variables measured once per animal, comparisons among multiple groups were performed using one-way ANOVA followed by Dunnett’s multiple-comparisons test when treatment groups were compared with the MPTP group, where the relevant assumptions were satisfied. A two-sided *p* < 0.05 was considered statistically significant for conventional analyses. Multiple-testing procedures for microbiome and metabolomics analyses are described in Section 2.9 and Section 2.10.

No single primary endpoint was prospectively designated before the experiment. Accordingly, behavioral, histological, RT-qPCR, and ELISA measurements were treated as planned study outcomes, whereas 16S rRNA sequencing, untargeted serum metabolomics, pathway-enrichment analyses, and descriptive cross-omics group-level visualization were considered exploratory analyses.

Because microbiota and serum metabolomic measurements were not completely paired at the individual-animal level, no inferential microbiota–metabolite correlation analysis was performed. Representative microbial and metabolic features were standardized independently within their respective datasets and summarized as group-level mean z-scores for descriptive visualization. This analysis was considered descriptive and hypothesis-generating only and was not used to infer individual-level associations or causal relationships.

## 3. Results

### 3.1. SHOU LAC-1 Was Associated with Attenuation of MPTP-Induced Reduction in Body-Weight Gain and Motor Impairment in Mice

The experimental design is presented in Figure 1A. Body weight was monitored throughout the experimental period to evaluate the general physiological status of mice during SHOU LAC-1 administration and MPTP modeling. As shown in Figure 1B, body weight gradually increased during the early phase of the experiment and tended to decline during the later modeling period. Because body weight was repeatedly measured in the same animals, longitudinal body-weight data were analyzed using a two-way repeated-measures analysis. The analysis showed no significant group effect (*p* = 0.1610) or group × time interaction (*p* = 0.1317), whereas the time effect was significant (*p* < 0.0001). Mice in the MPTP group showed significantly lower body-weight gain than the Control group (adjusted *p* = 0.0009; Figure 1C). Compared with the MPTP group, body-weight gain was significantly higher in both the L-DOPA (adjusted *p* = 0.0074) and SHOU LAC-1 groups (adjusted *p* < 0.0001).

Motor function was evaluated using the rotarod, pole, and balance beam tests. For each behavioral testing session, each mouse underwent three within-session trials, and the mean of the three trials was used as one outcome value for that animal. Individual within-session trials were not treated as independent observations. The three testing sessions performed in the same animals were analyzed using repeated-measures models.

In the rotarod test, mice in the MPTP group showed shorter latencies to fall than Control mice, whereas higher latencies were observed in the L-DOPA and SHOU LAC-1 groups (Figure 1D). These findings suggest that SHOU LAC-1 administration improved motor coordination. Repeated-measures analysis showed significant group and testing-session effects (both *p* < 0.0001), with no significant group × session interaction (*p* = 0.5234).

In the pole test, MPTP-treated mice showed longer descent times than Control mice, whereas lower descent times were observed in the L-DOPA and SHOU LAC-1 groups (Figure 1E). These findings suggest that SHOU LAC-1 may partly alleviate MPTP-associated bradykinesia-like behavioral impairment. Repeated-measures analysis showed significant group and testing-session effects (both *p* < 0.0001), with no significant group × session interaction (*p* = 0.8713).

In the balance beam test, mice in the MPTP group showed longer traversal times than Control mice, whereas lower traversal times were observed in the L-DOPA and SHOU LAC-1 groups (Figure 1F). These results suggest that SHOU LAC-1 intervention was associated with partial improvement in MPTP-induced balance and fine motor deficits. Repeated-measures analysis showed a significant group effect (*p* < 0.0001) and a significant group × session interaction (*p* = 0.0019), whereas the testing-session effect was not significant (*p* = 0.0554).

Collectively, MPTP treatment was associated with reduced body-weight gain and impaired motor performance, whereas SHOU LAC-1 administration was associated with attenuation of these MPTP-induced abnormalities.

### 3.2. SHOU LAC-1 Was Associated with Partial Preservation of Nigrostriatal Dopaminergic Markers and Increased Neurotrophic-Factor-Related Gene Expression

To further examine whether the behavioral improvement observed after SHOU LAC-1 administration was accompanied by changes in nigrostriatal dopaminergic markers, immunohistochemical staining for tyrosine hydroxylase (TH) and α-synuclein (α-SYN) was performed. TH immunoreactivity in the substantia nigra and striatum was assessed by quantifying TH-positive areas, whereas α-SYN immunoreactivity in the substantia nigra was evaluated by quantifying the α-SYN-positive area. In parallel, the striatal mRNA expression levels of TH, BDNF, and GDNF were measured to evaluate dopaminergic- and neurotrophic-support-related gene expression.

**Figure 2:**
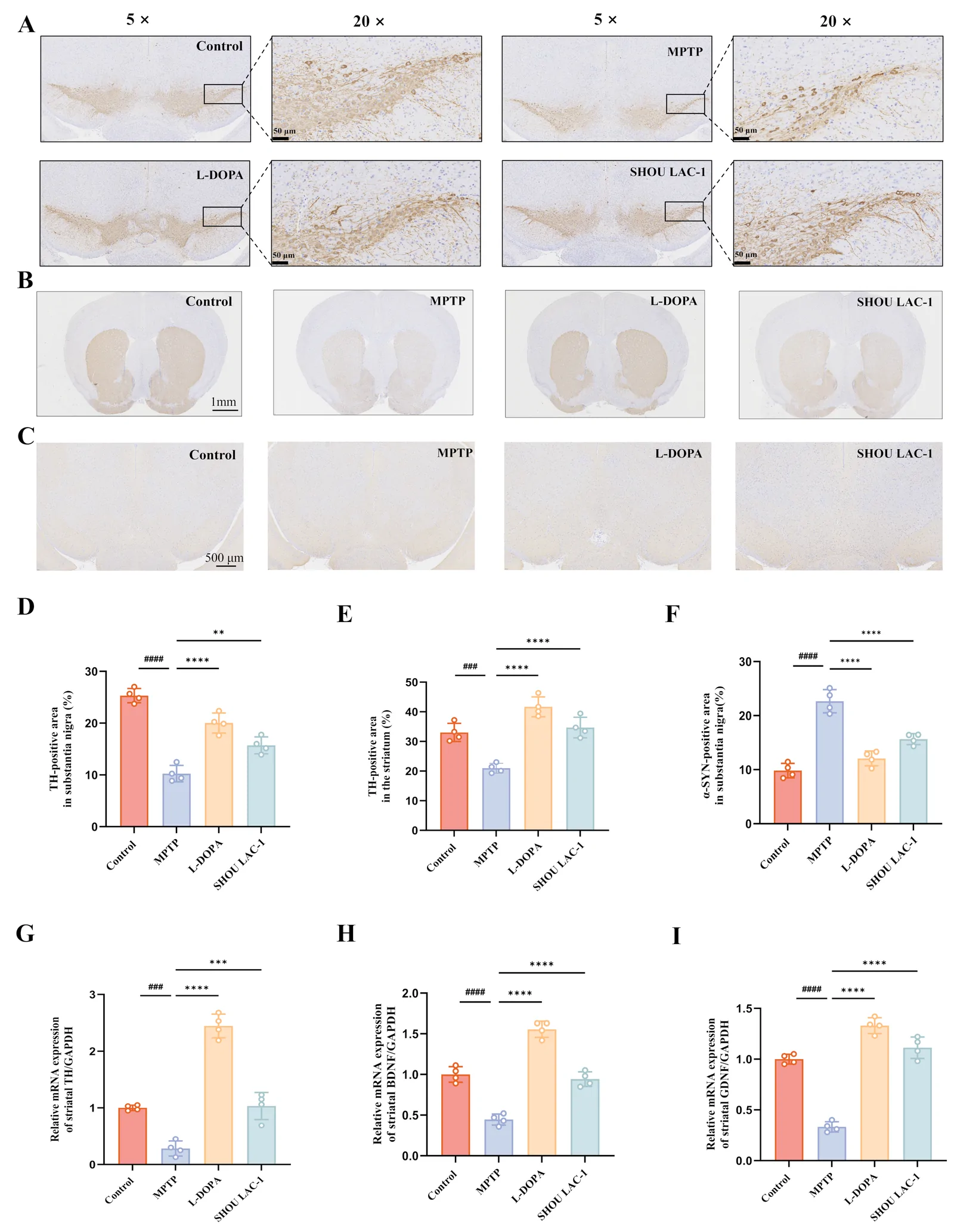
SHOU LAC-1 Was Associated with Partial Preservation of Nigrostriatal Dopaminergic Markers and Increased Neurotrophic-Factor-Related Gene Expression. (**A**) Representative immunohistochemical images showing TH immunoreactivity in the substantia nigra. (**B**) Representative immunohistochemical images showing TH immunoreactivity in the striatum. (**C**) Representative immunohistochemical images showing α-SYN immunoreactivity in the substantia nigra. (**D**) Quantification of the TH-positive area in the substantia nigra. (**E**) Quantification of the TH-positive area in the striatum. (**F**) Quantification of the α-SYN-positive area in the substantia nigra. (**G**–**I**) Relative mRNA expression levels of striatal TH, BDNF, and GDNF determined by RT-qPCR. For immunohistochemical analyses in panels (**A**–**F**), *n* = 4 biological samples per group; for RT-qPCR analyses in panels (**G**–**I**), *n* = 4 biological samples per group. Data are presented as the mean ± SD. ### *p* < 0.001 and #### *p* < 0.0001 versus the Control group; ** *p* < 0.01, *** *p* < 0.001, and **** *p* < 0.0001 versus the MPTP group.

As shown in Figure 2A–C, MPTP-treated mice exhibited visibly weaker TH-positive staining in the substantia nigra and striatum than Control mice, whereas α-SYN-positive staining in the substantia nigra was increased. Quantitative analysis confirmed that the TH-positive areas in both the substantia nigra and striatum were significantly reduced in the MPTP group compared with the Control group. In contrast, the α-SYN-positive area in the substantia nigra was significantly increased after MPTP treatment (Figure 2D–F). These results indicate that MPTP treatment was associated with reduced TH immunoreactivity in the substantia nigra and striatum and increased α-SYN immunoreactivity in the substantia nigra.

Compared with the MPTP group, L-DOPA treatment increased TH-positive areas in the substantia nigra and striatum and reduced α-SYN-positive staining in the substantia nigra. Compared with the MPTP group, SHOU LAC-1-treated mice showed larger TH-positive areas in the substantia nigra and striatum and a smaller α-SYN-positive area in the substantia nigra (Figure 2D–F). These findings indicate that SHOU LAC-1 administration was associated with partial preservation of TH immunoreactivity and reduced α-SYN immunoreactivity.

The expression of dopaminergic- and neurotrophic-support-related genes was then examined in the striatum. Compared with the Control group, MPTP treatment significantly decreased the relative mRNA expression levels of TH, BDNF, and GDNF (Figure 2G–I). L-DOPA treatment significantly increased the relative mRNA expression levels of TH, BDNF, and GDNF compared with the MPTP group. SHOU LAC-1 administration also significantly increased the relative mRNA expression levels of TH, BDNF, and GDNF compared with the MPTP group.

Collectively, MPTP treatment was associated with reduced TH immunoreactivity in the substantia nigra and striatum, increased α-SYN immunoreactivity in the substantia nigra, and decreased striatal expression of TH, BDNF, and GDNF. SHOU LAC-1 administration was associated with partial preservation of TH immunoreactivity, reduced α-SYN immunoreactivity, and increased striatal expression of TH, BDNF, and GDNF relative to the MPTP group. These findings suggest that the observed improvement in motor performance was accompanied by changes in nigrostriatal dopaminergic markers and neurotrophic-factor-related gene expression.

### 3.3. SHOU LAC-1 Partially Improved Defecatory Function and Colonic Barrier-Related Indicators

Given that gastrointestinal dysfunction is frequently observed as a non-motor manifestation of PD, defecatory function and colonic barrier-related indicators were further evaluated in MPTP-treated mice. Defecatory function was assessed by measuring the number of fecal pellets, total stool weight, and fecal water content within 30 min. Colonic morphology was examined by hematoxylin and eosin staining, and tight-junction-related changes were assessed by determining the mRNA expression levels of ZO-1, Occludin, and Claudin-1.

**Figure 3:**
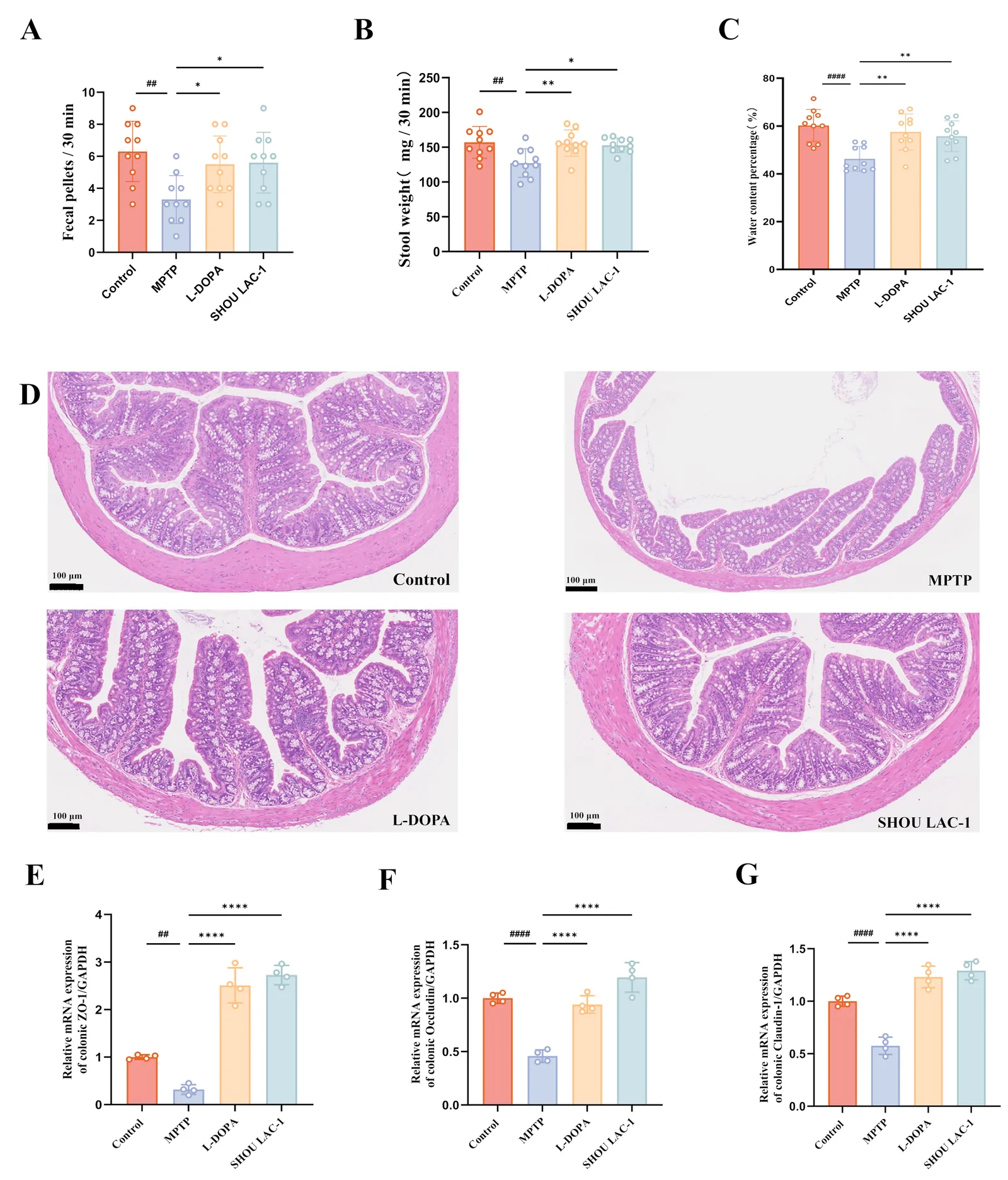
*Lactiplantibacillus plantarum* SHOU LAC-1 partially improved MPTP-associated defecatory dysfunction and colonic barrier-related indicators. (**A**) Number of fecal pellets within 30 min. (**B**) Total stool weight within 30 min. (**C**) Fecal water content. (**D**) Representative images of H&E-stained colon sections from each group. (**E**–**G**) Relative mRNA expression levels of the colonic tight-junction-related genes ZO-1, Occludin, and Claudin-1 determined by RT-qPCR. For defecatory measurements, *n* = 10 mice/group; for H&E staining, *n* = 4 biological samples/group; for RT-qPCR analyses, *n* = 4 biological samples/group. Data are presented as the mean ± SD. ## *p* < 0.01 and #### *p* < 0.0001 versus the Control group; * *p* < 0.05, ** *p* < 0.01, and **** *p* < 0.0001 versus the MPTP group.

As shown in Figure 3A–C, the number of fecal pellets, stool weight, and fecal water content was significantly reduced in the MPTP group compared with the Control group. These findings indicate that MPTP treatment was associated with impaired defecatory function. Compared with the MPTP group, these defecatory parameters were increased in the L-DOPA and SHOU LAC-1 groups, consistent with partial improvement of MPTP-associated defecatory dysfunction.

Histological examination showed that the colonic mucosa in the Control group displayed a relatively intact architecture, with regularly arranged glands and preserved mucosal organization. In contrast, descriptive examination of the MPTP group showed a less organized glandular arrangement and partial disruption of mucosal architecture. Compared with the MPTP group, the L-DOPA and SHOU LAC-1 groups showed comparatively better-preserved colonic morphology, including more orderly glandular structures and mucosal organization (Figure 3D). These observations were descriptive and were not based on a blinded quantitative histopathological scoring system.

The expression of tight-junction-related genes was then analyzed as an additional colonic barrier-related indicator. Compared with the Control group, the mRNA expression levels of colonic ZO-1, Occludin, and Claudin-1 were significantly decreased in the MPTP group (Figure 3E–G). Compared with the MPTP group, both L-DOPA and SHOU LAC-1 administration increased the expression of these tight-junction-related genes, with SHOU LAC-1 treatment being associated with increased ZO-1, Occludin, and Claudin-1 mRNA expression.

Collectively, MPTP treatment was associated with impaired defecatory function, altered colonic morphology, and decreased expression of tight-junction-related genes. SHOU LAC-1 administration partially improved defecatory parameters and was associated with comparatively better-preserved colonic morphology and increased expression of tight-junction-related genes. These findings therefore indicate improvements in colonic barrier-related indicators rather than direct evidence of restored intestinal barrier function.

### 3.4. SHOU LAC-1 Was Associated with Attenuated Inflammatory Responses in the Midbrain, Striatum, and Colon

To evaluate inflammatory responses following SHOU LAC-1 administration, representative pro-inflammatory cytokines were measured in central and peripheral tissues. The protein levels of TNF-α, IL-1β, and IL-6 were determined in the midbrain and colon by ELISA. In parallel, the mRNA expression levels of these inflammatory cytokines were analyzed in the striatum and colon. The striatal mRNA expression levels of GFAP and Iba-1 were also examined as glial-cell-related markers.

**Figure 4:**
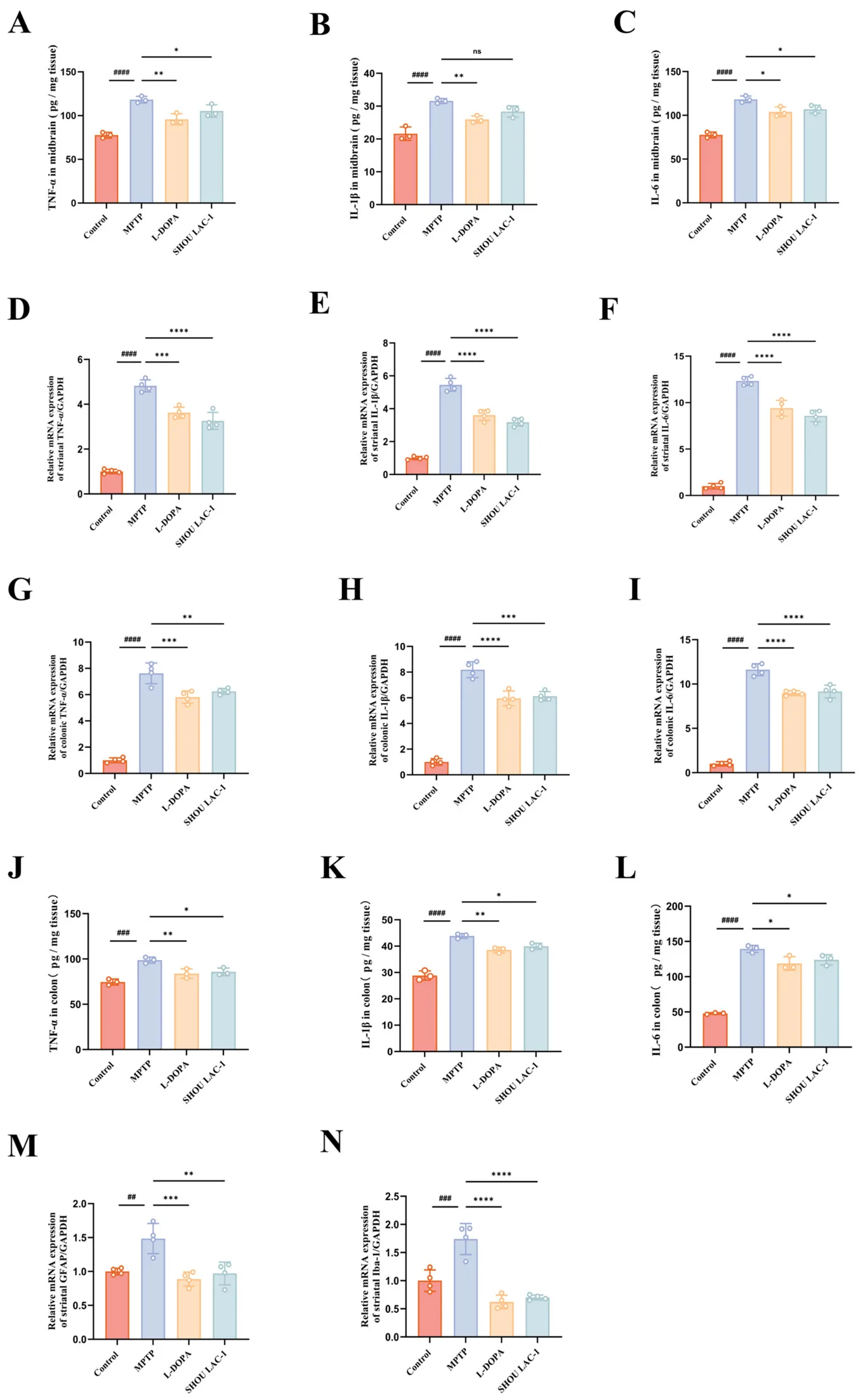
*Lactiplantibacillus plantarum* SHOU LAC-1 was associated with attenuated inflammatory responses in the midbrain, striatum, and colon. (**A**–**C**) Midbrain levels of TNF-α, IL-1β, and IL-6 measured by ELISA. (**D**–**F**) Relative mRNA expression levels of striatal TNF-α, IL-1β, and IL-6 determined by RT-qPCR. (**G**–**I**) Relative mRNA expression levels of colonic TNF-α, IL-1β, and IL-6 determined by RT-qPCR. (**J**–**L**) Colonic levels of TNF-α, IL-1β, and IL-6 measured by ELISA. (**M**,**N**) Relative mRNA expression levels of striatal GFAP and Iba-1 determined by RT-qPCR. For ELISA measurements in panels (**A**–**C**) and (**J**–**L**), *n* = 3 biological samples per group; for RT-qPCR analyses in panels (**D**–**I**) and (**M**,**N**), *n* = 4 biological samples per group. Data are presented as the mean ± SD. ## *p* < 0.01, ### *p* < 0.001, and #### *p* < 0.0001 versus the Control group; * *p* < 0.05, ** *p* < 0.01, *** *p* < 0.001, and **** *p* < 0.0001 versus the MPTP group; ns indicates no statistical significance.

As shown in Figure 4A–C, the midbrain levels of TNF-α, IL-1β, and IL-6 were significantly increased in the MPTP group compared with the Control group. These results indicate that MPTP treatment was associated with increased midbrain pro-inflammatory cytokine levels. Compared with the MPTP group, L-DOPA treatment reduced the levels of these cytokines. After SHOU LAC-1 administration, the levels of TNF-α and IL-6 were significantly decreased, whereas the difference in IL-1β was not statistically significant.

Striatal inflammation-related gene expression was then examined. Compared with the Control group, the mRNA expression levels of striatal TNF-α, IL-1β, and IL-6 were markedly upregulated in the MPTP group (Figure 4D–F). Compared with the MPTP group, both L-DOPA and SHOU LAC-1 treatment reduced the expression levels of these inflammatory cytokine genes. These findings suggest that SHOU LAC-1 was associated with attenuation of MPTP-induced inflammatory gene expression in the striatum.

A similar inflammatory pattern was observed in the colon. MPTP treatment increased the colonic mRNA expression levels of TNF-α, IL-1β, and IL-6 compared with those in the Control group. These increases were reduced after L-DOPA or SHOU LAC-1 administration (Figure 4G–I). Consistently, the colonic protein levels of TNF-α, IL-1β, and IL-6 were elevated in the MPTP group and were decreased in the L-DOPA and SHOU LAC-1 groups (Figure 4J–L). These results indicate that SHOU LAC-1 was associated with reduced colonic pro-inflammatory cytokine expression and protein levels.

The expression of glial-cell-related markers was further assessed in the striatum. Compared with the Control group, MPTP treatment significantly increased the mRNA expression levels of GFAP and Iba-1. Compared with the MPTP group, both L-DOPA and SHOU LAC-1 treatment decreased GFAP and Iba-1 mRNA expression (Figure 4M,N). Because glial-cell morphology and protein-level markers were not evaluated, these transcriptional changes should not be interpreted as direct evidence of suppressed astrocyte or microglial activation.

Collectively, MPTP treatment was associated with increased pro-inflammatory cytokine levels and/or gene expression in the midbrain, striatum, and colon, together with increased striatal expression of glial-cell-related markers. SHOU LAC-1 administration was associated with reductions in these inflammatory indicators and lower striatal expression of glial-cell-related markers.

### 3.5. SHOU LAC-1 Was Associated with Partial Recovery of Nrf2-Related Antioxidant Defense Gene Expression

To examine antioxidant-defense-related gene expression following SHOU LAC-1 intervention, the striatal mRNA expression levels of the antioxidant-defense-related genes Nrf2, NQO1, and HO-1 were examined. Nrf2 is a key transcription factor involved in cellular antioxidant responses, whereas NQO1 and HO-1 are representative downstream antioxidant-defense-related genes.

**Figure 5:**
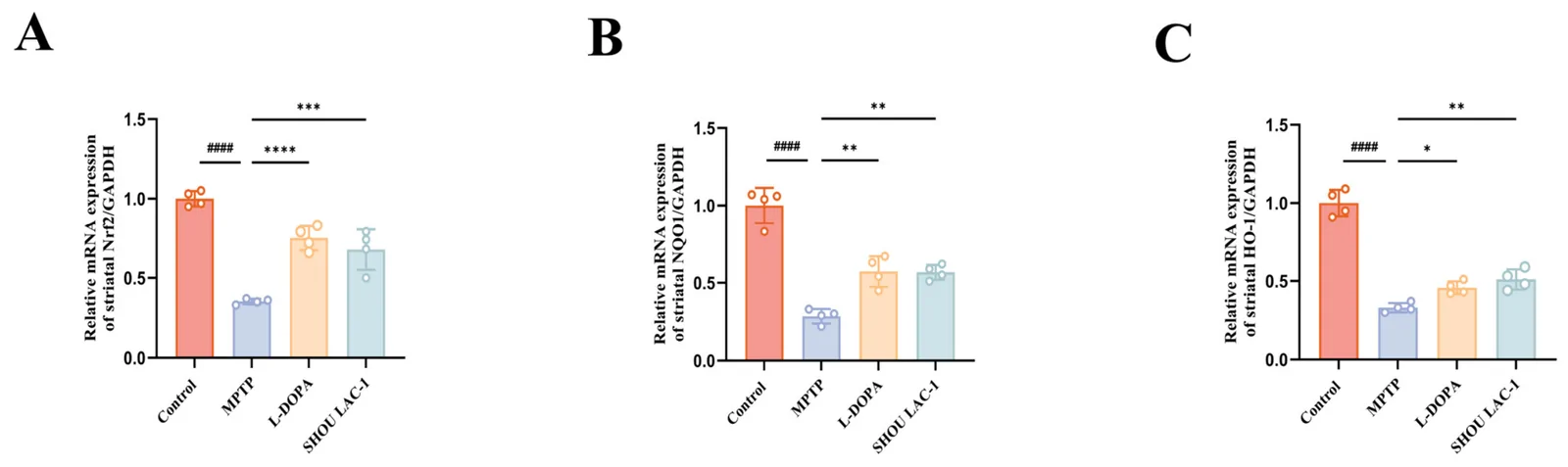
*Lactiplantibacillus plantarum* SHOU LAC-1 was associated with partial recovery of Nrf2-related antioxidant-defense gene expression in the striatum of MPTP-treated mice. (**A**–**C**) Relative mRNA expression levels of striatal Nrf2, NQO1, and HO-1 determined by RT-qPCR. *n* = 4 biological samples per group. Data are presented as the mean ± SD. #### *p* < 0.0001 versus the Control group; * *p* < 0.05, ** *p* < 0.01, *** *p* < 0.001, and **** *p* < 0.0001 versus the MPTP group.

As shown in Figure 5A–C, the mRNA expression levels of striatal Nrf2, NQO1, and HO-1 were significantly decreased in the MPTP group compared with the Control group. These results suggest that MPTP treatment was associated with reduced expression of Nrf2-related antioxidant-defense genes in the striatum. Compared with the MPTP group, L-DOPA treatment increased the expression levels of these genes.

After SHOU LAC-1 administration, the mRNA expression levels of Nrf2, NQO1, and HO-1 were also higher than those in the MPTP group (Figure 5A–C). Although the extent of recovery differed among these genes, SHOU LAC-1 treatment was associated with partial recovery of Nrf2-related antioxidant-defense gene expression.

Collectively, MPTP treatment was associated with decreased striatal Nrf2, NQO1, and HO-1 mRNA expression, whereas higher expression levels were observed following SHOU LAC-1 administration. These findings indicate partial recovery of antioxidant-defense-related gene expression; however, they do not demonstrate functional restoration of the antioxidant defense system or a reduction in oxidative stress.

### 3.6. SHOU LAC-1 Administration Was Associated with Changes in Gut Microbial Diversity and Composition

**Figure 6:**
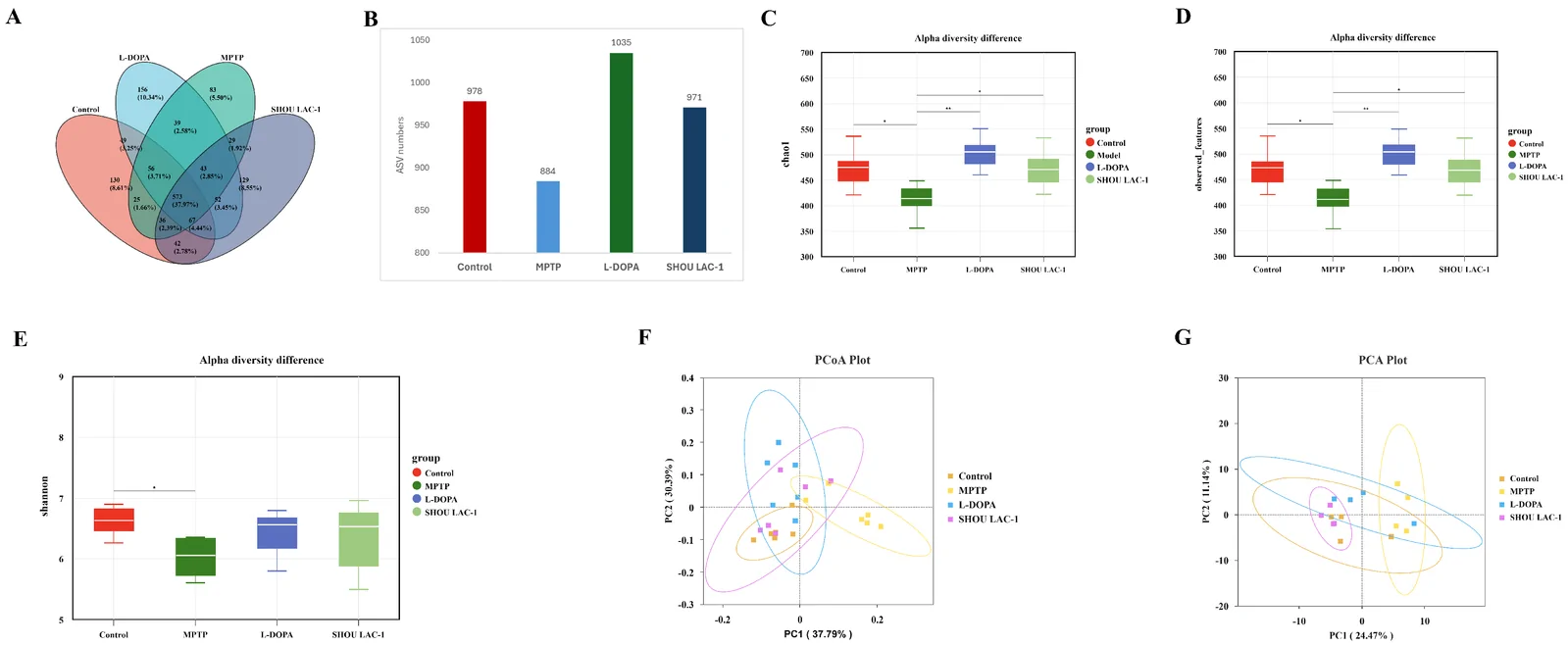
ASV composition and microbial diversity analysis of the gut microbiota in the Control, MPTP, L-DOPA, and SHOU LAC-1 groups. (**A**) Venn diagram showing shared and group-specific ASVs among the four groups. (**B**) Total number of ASVs detected in each group. (**C**–**E**) α-diversity analysis, including the Chao1 index, Observed features index, and Shannon index. (**F**) PCoA of β-diversity. (**G**) PCA of β-diversity. *n* = 6 biological samples per group. Statistical significance for the indicated pairwise comparisons is shown above the corresponding brackets: * *p* < 0.05 and ** *p* < 0.01.

To determine whether SHOU LAC-1 administration was associated with changes in the gut microbiota of MPTP-treated mice, fecal samples were analyzed by 16S rRNA gene sequencing. As shown in Figure 6A,B, shared and group-specific amplicon sequence variants (ASVs) were detected among the four groups. The total number of ASVs was reduced in the MPTP group compared with the Control group, whereas higher ASV numbers were observed in the L-DOPA and SHOU LAC-1 groups. These observations indicate differences in the numbers of detected ASVs among the experimental groups.

According to the α-diversity analysis, the Chao1, Observed features, and Shannon indices were significantly lower in the MPTP group compared with the Control group (Figure 6C–E). These findings indicate significant differences in α-diversity indices between the Control and MPTP groups. Compared with the MPTP group, the Chao1 and Observed features indices were significantly higher in the L-DOPA and SHOU LAC-1 groups, while the Shannon index did not differ significantly between the MPTP and SHOU LAC-1 groups.

PCoA and PCA showed different sample-distribution patterns among the experimental groups (Figure 6F,G). PERMANOVA (Adonis) showed a significant difference in microbial community composition between the Control and MPTP groups (pseudo-*F* = 2.568, R^2^ = 0.125, *p* = 0.002) and between the MPTP and SHOU LAC-1 groups (pseudo-*F* = 1.984, R^2^ = 0.099, *p* = 0.017). These results indicate differences in microbial community composition between the relevant experimental groups.

**Figure 7:**
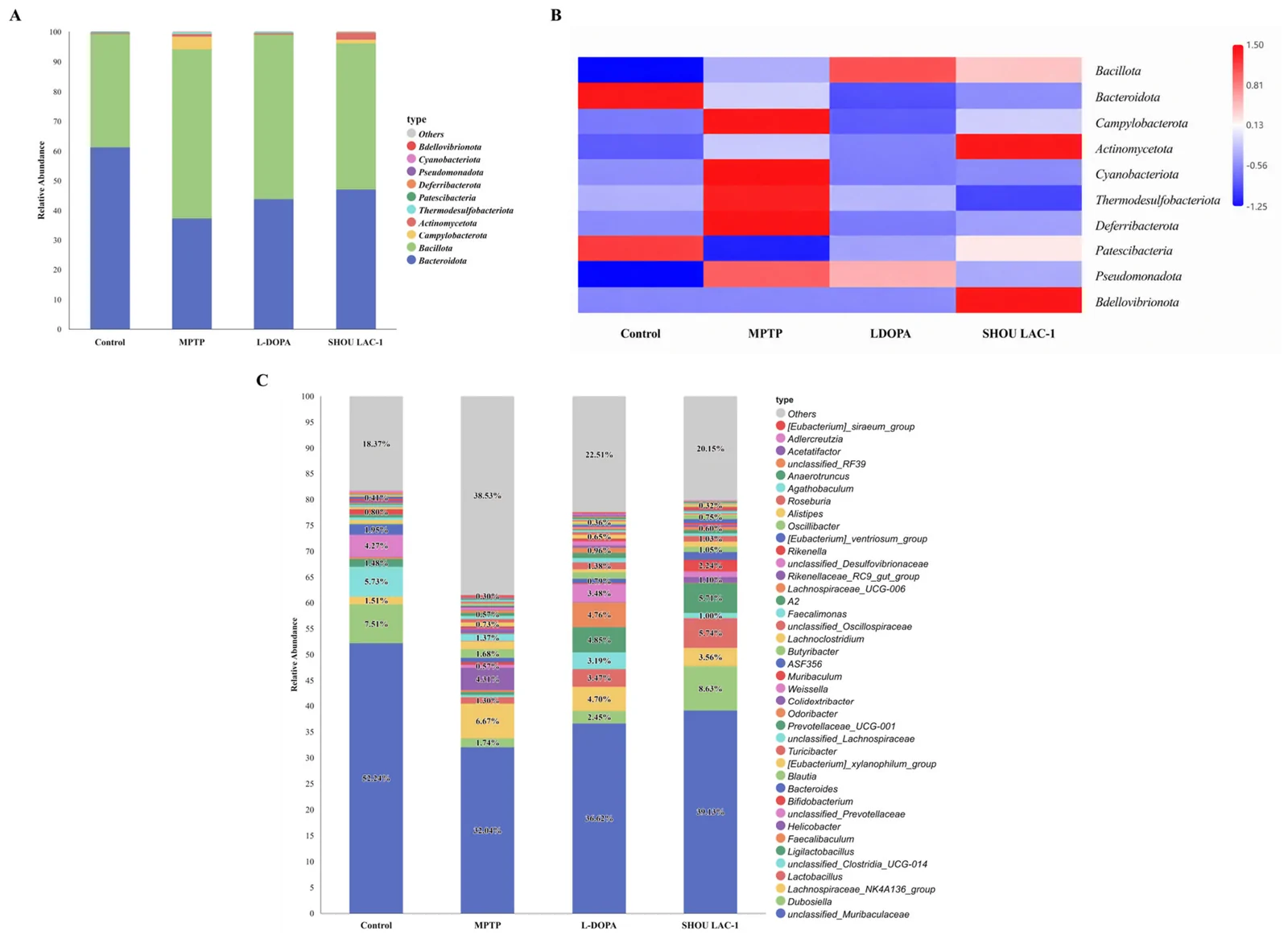
Phylum- and genus-level composition of the gut microbiota in the Control, MPTP, L-DOPA, and SHOU LAC-1 groups. (**A**) Stacked bar plot showing the relative abundance of bacterial taxa at the phylum level in individual samples from the four groups. (**B**) Cluster heatmap of the relative abundance of dominant bacterial phyla. (**C**) Stacked bar plot showing the relative abundance of bacterial taxa at the genus level in individual samples from the four groups. *n* = 6 biological samples per group. Heatmap colors indicate normalized relative abundance, with red representing higher relative abundance and blue representing lower relative abundance.

The taxonomic composition of the gut microbiota was then examined at the phylum and genus levels. At the phylum level, *Bacteroidota* and *Bacillota* were the predominant bacterial phyla across all groups (Figure 7A,B). Compared with the Control group, the relative abundance of *Bacteroidota* was lower in the MPTP group, whereas *Bacillota* was higher. Relative to the MPTP group, the SHOU LAC-1 group showed a higher relative abundance of *Bacteroidota* and a lower relative abundance of *Bacillota*. These observations indicate differences in the relative abundance profiles of the dominant bacterial phyla among the experimental groups.

At the genus level, MPTP administration was accompanied by differences in the gut microbial composition compared with the Control group (Figure 7C). Unclassified_Muribaculaceae was a predominant taxon in the Control group and showed a lower relative abundance in the MPTP group, whereas the proportion of low-abundance taxa grouped as “Others” was higher. The L-DOPA and SHOU LAC-1 groups showed different genus-level relative abundance profiles from the MPTP group.

In addition, MPTP treatment was accompanied by changes in several taxa, including *Bifidobacterium*, *Lactobacillus*, *Faecalibaculum*, *Turicibacter*, and unclassified_Desulfovibrionaceae. Compared with the MPTP group, differences were observed in several taxa in the SHOU LAC-1 group, including Prevotellaceae_UCG-001, unclassified_Ruminococcaceae, unclassified_Oscillospiraceae, *Anaerotruncus*, and *Colidextribacter*. These observations indicate differences in genus-level microbial composition between the MPTP and SHOU LAC-1 groups [10,42].

**Figure 8:**
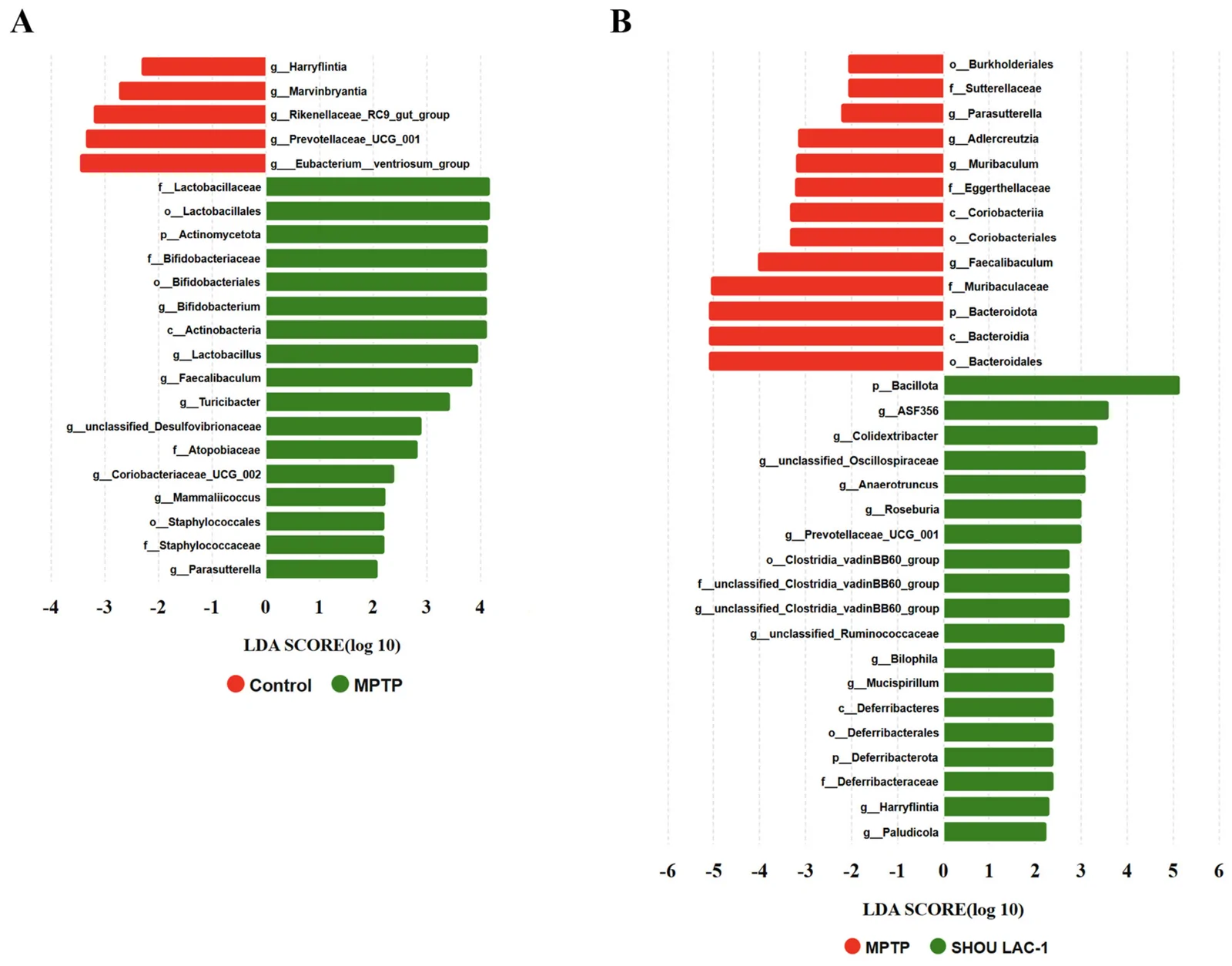
LEfSe analysis of differentially abundant gut microbial taxa in the Control, MPTP, and SHOU LAC-1-related comparisons. (**A**) LEfSe analysis of differentially abundant taxa between the Control and MPTP groups. (**B**) LEfSe analysis of differentially abundant taxa between the MPTP and SHOU LAC-1 groups. *n* = 6 biological samples per group. The bar plots show taxa identified by linear discriminant analysis, and LDA scores indicate the effect size of each differentially abundant taxon.

LEfSe analysis was further performed to identify differentially abundant bacterial taxa. Compared with the Control group, the MPTP group was mainly enriched in *Bifidobacterium*, *Lactobacillus*, *Faecalibaculum*, *Turicibacter*, unclassified Desulfovibrionaceae, and *Parasutterella*, whereas the Control group was mainly enriched in Prevotellaceae_UCG-001, Rikenellaceae_RC9_gut_group, *Harryflintia*, and [Eubacterium]_ventriosum_group (Figure 8A). These findings indicate differences in gut microbial taxonomic composition between the Control and MPTP groups.

Compared with the MPTP group, the SHOU LAC-1 group showed higher relative abundances of Prevotellaceae_UCG-001, unclassified Oscillospiraceae, unclassified Ruminococcaceae, *Anaerotruncus*, *Colidextribacter*, and *ASF356* (Figure 8B). These findings indicate compositional differences in the gut microbiota after SHOU LAC-1 administration.

Collectively, MPTP treatment was associated with differences in gut microbial diversity, community composition, and taxonomic profiles relative to the Control group, whereas SHOU LAC-1 administration was accompanied by differences in these microbial features relative to the MPTP group.

### 3.7. SHOU LAC-1 Administration Was Associated with Changes in Serum Metabolic Profiles

**Figure 9:**
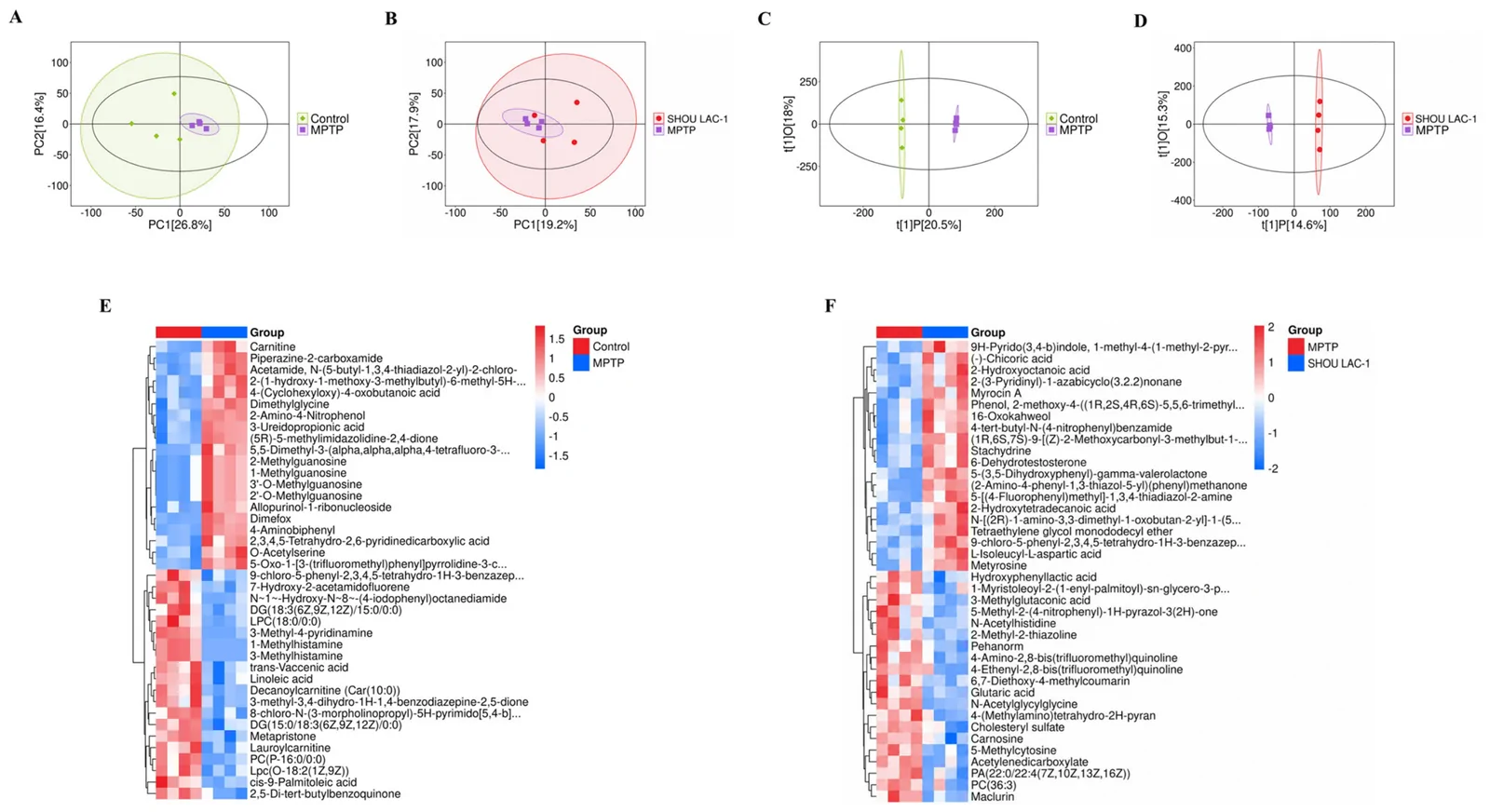
Serum metabolic profiles associated with MPTP treatment and SHOU LAC-1 administration. (**A**,**B**) PCA score plots comparing the Control and MPTP groups (**A**) and the MPTP and SHOU LAC-1 groups (**B**). (**C**,**D**) OPLS-DA score plots comparing the Control and MPTP groups (**C**) and the MPTP and SHOU LAC-1 groups (**D**). OPLS-DA models were evaluated using seven-fold cross-validation and 200 permutation tests. (**E**,**F**) Heatmaps showing representative candidate serum metabolic features for the Control versus MPTP comparison (**E**) and the MPTP versus SHOU LAC-1 comparison (**F**). Candidate features were initially screened using VIP > 1 and nominal *p* < 0.05, followed by Benjamini–Hochberg FDR correction. Corresponding fold-change values, individual VIP scores, raw *p*-values, FDR-adjusted q-values, and identification-confidence levels for the features shown in panels (**E**,**F**) are provided in Supplementary Table S2. Colors indicate normalized relative abundance. *n* = 4 biological samples per group in the final metabolomic analysis. PCA, principal component analysis; OPLS-DA, orthogonal partial least-squares discriminant analysis.

To characterize serum metabolic differences associated with MPTP treatment and SHOU LAC-1 administration, untargeted serum metabolomics was performed using four biological samples per group in the final metabolomic analysis. PCA showed differences in sample-distribution patterns between the Control and MPTP groups (Figure 9A). The distribution of samples from the SHOU LAC-1 group also differed from that of the MPTP group (Figure 9B).

OPLS-DA was further performed as an exploratory supervised analysis. Model performance and potential overfitting were evaluated using seven-fold cross-validation and 200 permutation tests. For the Control versus MPTP comparison, the model showed R^2^X(cum) = 0.384, R^2^Y(cum) = 1.000, and Q^2^(cum) = 0.625, and the permutation test supported the model (*p* < 0.05). For the MPTP versus SHOU LAC-1 comparison, R^2^X(cum) = 0.299, R^2^Y(cum) = 0.999, and Q^2^(cum) = 0.353; however, the permutation test was not significant (*p* > 0.05) (Figure 9C,D). Therefore, although an apparent separation was observed in the MPTP versus SHOU LAC-1 OPLS-DA score plot, this separation was interpreted cautiously because the corresponding model was not supported by permutation testing.

The heatmap showed group-associated abundance patterns among several candidate serum metabolic features. Compared with the Control group, candidate features annotated as Carnitine, 2-Methylguanosine, 1-Methylguanosine, 3′-O-Methylguanosine, and 2′-O-Methylguanosine showed higher relative abundances in the MPTP group, whereas linoleic acid, trans-vaccenic acid, cis-9-palmitoleic acid, LPC(18:0/0:0), LPC(O-18:2(1Z,9Z)), PC(P-16:0/0:0), decanoylcarnitine, and lauroylcarnitine showed lower relative abundances (Figure 9E). These representative features included fatty-acid-, glycerophospholipid-, acylcarnitine-, and nucleotide-related compounds. Because these representative features did not remain significant after FDR correction, their differences were considered exploratory. Acylcarnitines are involved in mitochondrial fatty-acid transport and oxidation [43]. Comprehensive metabolite resources such as HMDB provide reference information for the nomenclature and classification of such serum metabolites [44].

Following SHOU LAC-1 administration, several representative candidate metabolic features showed different relative abundance patterns compared with the MPTP group. 2-Hydroxytetradecanoic acid and 2-hydroxyoctanoic acid were among the features showing higher relative abundance, whereas N-acetylhistidine, carnosine, cholesteryl sulfate, glutaric acid, and PC(36:3) showed lower relative abundance (Figure 9F). However, these representative features did not remain significant after FDR correction and were therefore interpreted as exploratory candidate metabolic features rather than statistically confirmed differential metabolites. Detailed feature-level quantitative information for the metabolic features shown in Figure 9E,F, including fold change, VIP score, raw *p*-value, FDR-adjusted q-value, and identification-confidence level, is provided in Supplementary Table S2.

**Figure 10:**
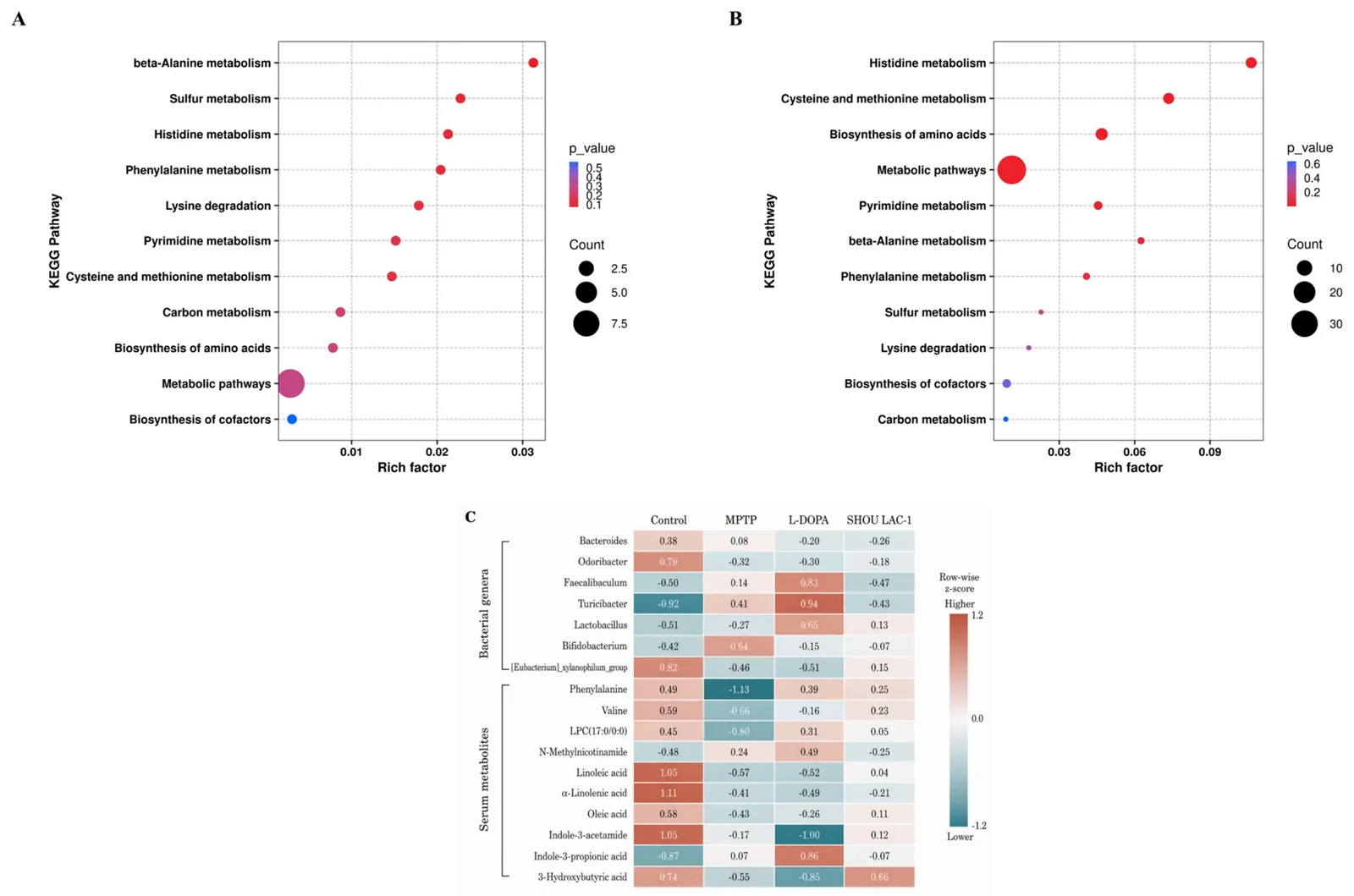
KEGG pathway-enrichment analysis of candidate serum metabolic features and descriptive group-level patterns of gut bacterial genera and serum metabolic features. (**A**) KEGG enrichment bubble plot for the Control versus MPTP comparison. (**B**) KEGG enrichment bubble plot for the MPTP versus SHOU LAC-1 comparison. Bubble size represents the number of metabolic features mapped to each pathway, and color indicates the nominal *p* value. Benjamini–Hochberg FDR correction was additionally applied for multiple testing, and FDR-adjusted results are reported in the Results. KEGG, Kyoto Encyclopedia of Genes and Genomes. (**C**) Descriptive heatmap showing group-level mean z-score patterns of representative gut bacterial genera and serum metabolic features across the Control, MPTP, L-DOPA, and SHOU LAC-1 groups. Microbiota data (*n* = 6 biological samples per group) and metabolomics data (*n* = 4 biological samples per group) were standardized independently within their respective datasets and summarized at the group level. Because the datasets were not completely paired at the individual-animal level, panel (**C**) is descriptive and hypothesis-generating only; no inferential correlation coefficients or *p*-values were calculated.

KEGG pathway-enrichment analysis [45] was further conducted using the candidate metabolic features, with Benjamini–Hochberg FDR correction applied for multiple testing. In the Control versus MPTP comparison, histidine metabolism (q = 0.0013), cysteine and methionine metabolism (q = 0.0037), and pyrimidine metabolism (q = 0.0499) remained significant after FDR correction (Figure 10A). Other pathways identified at the nominal p-value level, including β-alanine metabolism, phenylalanine metabolism, sulfur metabolism, and carbon metabolism, did not remain significant after FDR correction and were therefore considered exploratory. In the MPTP versus SHOU LAC-1 comparison, several pathways were identified at the nominal *p*-value level; however, no pathway remained significant after Benjamini–Hochberg FDR correction (Figure 10B). Accordingly, pathway-level findings for the SHOU LAC-1 comparison were interpreted as exploratory and were not considered evidence of confirmed pathway modulation or altered metabolic flux.

Collectively, the untargeted serum metabolomic analysis showed differences in serum metabolic profiles associated with MPTP treatment and SHOU LAC-1 administration. The candidate features included lipid-, amino-acid-, nucleotide-, and acylcarnitine-related compounds; however, most of the representative heatmap-selected features did not remain significant after FDR correction, and no KEGG pathway in the MPTP versus SHOU LAC-1 comparison remained significant after FDR correction. Therefore, the SHOU LAC-1-associated metabolomic findings should be considered exploratory and associative and do not establish specific metabolic remodeling or a causal metabolic mechanism.

### 3.8. Group-Level Patterns of Gut Microbial and Serum Metabolic Alterations

To descriptively compare gut microbial and serum metabolic patterns across the experimental groups, representative bacterial genera from the final 16S rRNA sequencing dataset (*n* = 6 biological samples per group) and representative serum metabolic features from the final untargeted metabolomics dataset (*n* = 4 biological samples per group) were visualized at the group level. Each selected feature was standardized within its corresponding dataset and summarized as the mean z-score for the Control, MPTP, L-DOPA, and SHOU LAC-1 groups (Figure 10C).

The resulting heatmap showed distinct group-level patterns among the experimental groups. However, because the microbiota and metabolomics measurements were not completely paired at the individual-animal level, these patterns were considered descriptive and hypothesis-generating only. No inferential microbiota–metabolite correlation coefficients or *p*-values were calculated. Accordingly, Figure 10C does not establish individual-level associations or causal relationships between specific bacterial taxa and serum metabolites.

## 4. Discussion

The present study evaluated *Lactiplantibacillus plantarum* SHOU LAC-1 as a strain-specific candidate in an MPTP-induced PD-like mouse model using an integrated assessment of central and peripheral outcomes. SHOU LAC-1 administration was associated with coordinated changes in motor and defecatory performance, nigrostriatal TH and α-SYN immunoreactivity, neurotrophic-support-related gene expression, inflammatory markers, glial-cell-related gene expression, Nrf2-related antioxidant gene expression, colonic morphology and tight-junction-related gene expression, gut microbial composition, and serum metabolic profiles. Inflammatory and glial-cell-related abnormalities are well-recognized components of PD pathophysiology [46,47]. The integrated evaluation of neurological, gastrointestinal, inflammatory, antioxidant-related, microbial, and metabolic outcomes represents a distinctive aspect of the present study. However, these findings are predominantly preclinical and associative and do not establish a causal microbiota–gut–brain pathway. Moreover, because SHOU LAC-1 administration began before MPTP exposure and continued during the modeling period, the present design primarily evaluates preventive/protective attenuation of MPTP-induced abnormalities rather than therapeutic reversal of established PD-like pathology.

MPTP is widely used to induce PD-like dopaminergic and motor abnormalities because its active metabolite, MPP+, disrupts mitochondrial function and damages the nigrostriatal dopaminergic system [30,31,32,48,49]. In the present study, MPTP treatment was accompanied by smaller TH-positive areas in the substantia nigra and striatum and greater α-SYN immunoreactivity in the substantia nigra, whereas SHOU LAC-1 administration was associated with partial preservation of TH immunoreactivity and lower α-SYN immunoreactivity. These histological findings were accompanied by improved motor performance. Nevertheless, TH immunoreactivity is an indirect indicator of dopaminergic integrity, and the present analysis quantified TH-positive area rather than stereologically determined dopaminergic neuronal number. Likewise, α-SYN-positive area does not distinguish among soluble, oligomeric, and aggregated forms of α-SYN. The findings therefore support treatment-associated differences in TH and α-SYN immunoreactivity rather than definitive neuronal rescue or reduced α-SYN aggregation.

Striatal *Bdnf* and *Gdnf* mRNA expression was lower after MPTP treatment and higher following SHOU LAC-1 administration. These genes are relevant to neurotrophic support of the dopaminergic system [50,51]; however, their assessment in the present study was restricted to the transcriptional level. Accordingly, the findings indicate changes in neurotrophic-support-related gene expression but do not demonstrate restoration of BDNF- or GDNF-mediated protein signaling. Taken together with the TH and α-SYN findings, the results suggest coordinated changes in dopaminergic- and neurotrophic-support-related indicators, while the underlying molecular mechanisms remain to be established.

Gastrointestinal abnormalities represent an important peripheral component of the PD-like phenotype examined in this study [6,7,52,53,54]. MPTP-treated mice showed reduced fecal pellet number, fecal weight, and water content, together with histomorphological alterations in the colon and lower mRNA expression of the tight-junction-related genes *ZO-1*, *Occludin*, and *Claudin-1*. SHOU LAC-1 administration was associated with improved defecatory parameters, comparatively better-preserved colonic morphology, and increased expression of these tight-junction-related genes. However, epithelial permeability and tight-junction protein abundance and localization were not directly assessed, and microbial translocation was not measured. Therefore, these observations should be interpreted as changes in gastrointestinal and intestinal barrier-related indicators rather than direct evidence of restored intestinal barrier function. Prior PD studies have linked increased intestinal permeability with intestinal α-synuclein and endotoxin-exposure markers [55], but those functional permeability and translocation endpoints were not directly evaluated here.

Inflammatory changes were observed in both central and colonic tissues. SHOU LAC-1 administration was associated with lower levels or expression of several inflammatory markers relative to MPTP treatment. These findings are consistent with an association between SHOU LAC-1 administration and a less pronounced inflammatory-marker profile in the tissues examined. However, they do not establish a causal inflammatory pathway connecting the intestine and brain, because circulating inflammatory mediators, microbial translocation, and blood–brain barrier permeability were not evaluated. In addition, GFAP and Iba-1 were measured at the mRNA level. Their lower expression after SHOU LAC-1 administration should therefore be described as changes in glial-cell-related gene expression rather than direct evidence of suppression of astrocyte or microglial activation. Protein-level and cell-specific morphological analyses would be required to characterize glial activation directly. These observations are consistent with the established relevance of inflammatory and glial-cell-related abnormalities to PD and MGBA-related neuroinflammatory processes [46,47,53,54].

MPTP treatment was also accompanied by lower striatal expression of Nrf2-, HO-1-, and NQO1-related genes, whereas higher expression was observed following SHOU LAC-1 administration. These results indicate changes in Nrf2-related antioxidant gene expression. Nrf2-related antioxidant signaling has been implicated in MPTP-induced injury and broader neurodegenerative oxidative-stress responses [56,57,58]. Nevertheless, Nrf2 nuclear translocation, HO-1 and NQO1 protein abundance, antioxidant enzyme activity, reactive oxygen species, and direct oxidative-damage markers were not measured. Consequently, the present findings should not be interpreted as direct evidence of Nrf2 pathway activation, restoration of antioxidant capacity, or functional reduction in oxidative stress. Protein-level and functional validation will be required to determine the biological significance of these transcriptional changes.

The gut microbiota data further showed that SHOU LAC-1 administration was associated with changes in microbial diversity and taxonomic composition relative to MPTP treatment. Community-level ordination analyses showed group-related distribution patterns in microbial composition, but these findings should not be interpreted as restoration of a normal microbial state or as evidence of altered microbial metabolic activity. Several taxa showing treatment-associated differences, including members of *Ruminococcaceae*-, *Oscillospiraceae*-, and *Prevotellaceae*-related groups, have previously been associated in the literature with fermentation or SCFA-related functions [10,12]. However, 16S rRNA sequencing provides limited taxonomic and functional resolution, and the presence or relative abundance of a bacterial taxon does not establish its metabolic activity in vivo. Most importantly, acetate, propionate, butyrate, valerate, and other SCFAs were not directly quantified in the present study. Therefore, references to SCFA-related taxa are based solely on taxonomic composition and previously reported biological associations and should be regarded as hypothesis-generating rather than evidence that SHOU LAC-1 increased SCFA production, restored SCFA-associated microbial function, or mediated its effects through SCFAs. In addition, increases or decreases in *Lactobacillus*, *Bifidobacterium*, and other genus-level taxa should not be interpreted as uniformly beneficial or detrimental because their biological functions are species-, strain-, and context-dependent. Functional metagenomic analyses together with direct targeted measurement of microbial metabolites would be required to test these possibilities. The *Muribaculaceae*-related differences observed in this dataset are also relevant to prior descriptions of this family’s metabolic and gut-homeostatic functions [59]. Desulfovibrio-related bacteria have been associated with PD-related gut dysbiosis and α-synuclein phenotypes in prior studies [60,61]. SCFA-related microbiota have also been discussed in relation to PD-associated neuroinflammatory and oxidative processes [62]. These interpretive cautions are consistent with the broader strain-specific probiotic literature [10,42,63].

Untargeted serum metabolomics showed that SHOU LAC-1 administration was accompanied by changes in several serum metabolic features relative to MPTP treatment. The observed patterns involved features related to fatty-acid, phospholipid, acylcarnitine, nucleotide, and other metabolic categories. However, these findings should be regarded as exploratory rather than as evidence of definitive metabolic remodeling. OPLS-DA was used as an exploratory supervised approach and was evaluated by cross-validation and permutation testing; nevertheless, supervised discrimination does not itself establish a biological mechanism. Furthermore, not all candidate metabolic features retained statistical significance after false-discovery-rate correction. Accordingly, features that did not remain significant after multiple-testing correction should be considered exploratory signals, and metabolite identities and concentration changes derived from untargeted analysis require targeted quantitative confirmation before specific metabolic pathways can be considered established. Comparable PD studies have reported alterations in amino-acid, lipid, nucleotide/purine, and microbiota-linked systemic metabolic features [17,18,19,20,64,65,66].

The descriptive group-level comparison of selected gut microbial and serum metabolic features should be interpreted with particular caution. Because the microbiota and serum metabolomics datasets were not completely paired one-to-one at the individual-animal level, the observed patterns cannot be interpreted as inferential individual-level microbiota–metabolite correlations. Instead, they represent descriptive and hypothesis-generating group-level patterns among selected microbial and metabolic features. Such patterns may be useful for generating hypotheses for future paired multi-omics studies, but the present data do not demonstrate direct microbiota–metabolite coupling, microbial production of particular serum metabolites, metabolite-mediated neurological effects, or directionality among microbial, intestinal, metabolic, and neurological changes. Thus, the current findings support parallel changes in multiple microbiota–gut–brain-axis-related readouts rather than a demonstrated causal microbiota–metabolite–brain mechanism. Previous PD multi-omics studies have likewise reported associations between gut microbial composition and systemic metabolic features [17,65,67], while gut microbiota-derived tryptophan/indole metabolites are biologically relevant to inflammatory, oxidative, and nervous-system homeostasis [68]. These prior observations provide biological context only and do not validate individual-level microbiota–metabolite coupling in the present dataset.

Taken together, the findings indicate coordinated central and peripheral changes following SHOU LAC-1 administration. Nevertheless, the present experimental design does not establish whether alterations in the intestinal environment, gut microbial composition, systemic metabolism, inflammatory and antioxidant-related indicators, and neurological outcomes are mechanistically linked. The results are therefore more appropriately interpreted as a multilevel association study within a microbiota–gut–brain axis framework rather than as direct elucidation of a causal pathway. Experimental PD-model studies have shown that gut-directed microbial or metabolite interventions can modify gastrointestinal, inflammatory, motor, or neurodegeneration-related outcomes [69,70,71,72,73,74]; however, the present design does not establish that such a causal pathway underlies the observed SHOU LAC-1-associated changes.

L-DOPA was included as a positive control to demonstrate the responsiveness of the experimental model; clinically, levodopa remains a standard symptomatic dopaminergic therapy [3,4]. Although SHOU LAC-1 and L-DOPA were associated with changes in partially overlapping outcomes, their administration schedules differed and the study was not designed as a superiority, equivalence, or non-inferiority comparison. No conclusion regarding their relative efficacy can therefore be drawn. SHOU LAC-1 should instead be regarded as a strain-specific candidate for further investigation as an adjunctive nutritional intervention and not as an alternative to standard dopaminergic therapy. This positioning is consistent with literature describing gut microbiota-targeted and synbiotic approaches as adjunctive rather than replacement strategies [75,76,77].

Several limitations should be acknowledged. First, the study included only young male mice and used a single MPTP-based PD-like model, which limits generalization across sexes, ages, and complementary experimental models. Second, a SHOU LAC-1-only control group was not included. Third, several mechanism-related outcomes were evaluated predominantly at the transcriptional level without corresponding protein-level or functional confirmation. In particular, intestinal permeability, tight-junction protein abundance and localization, Nrf2 nuclear translocation and downstream protein expression, direct oxidative-stress and antioxidant-capacity markers, glial-cell morphology and protein expression, aggregated α-SYN, circulating microbial products, and SCFA concentrations were not directly evaluated. The study also did not include causal microbiota-transfer or depletion experiments of the type used to test microbiota-dependent PD-like phenotypes in prior models [78]. Fourth, 16S rRNA sequencing provides limited strain-level and functional resolution [79], whereas untargeted metabolite annotations and abundance patterns require targeted quantitative validation. Fifth, incomplete individual-level pairing between the microbiota and metabolomics datasets precluded valid inferential microbiota–metabolite correlation analysis and therefore restricted the cross-omics comparison to descriptive group-level visualization. In addition, no formal a priori power calculation was performed and formal blinding was not implemented during behavioral testing or tissue collection, which should be considered when interpreting the findings. Future studies should therefore incorporate complementary PD models and sex/age groups, appropriate strain-only controls, individually paired multi-omics datasets, targeted SCFA and metabolite quantification, protein-level and functional assessment of intestinal barrier and antioxidant-related outcomes, and causal approaches such as microbiota transfer, microbiota depletion, germ-free models, or defined microbial/metabolite supplementation; validation across complementary chemically induced PD models is also warranted [11,62,80,81,82].

Overall, SHOU LAC-1 administration was associated with coordinated changes in central and peripheral indicators in MPTP-treated mice. These findings provide preliminary preclinical support for further strain-specific investigation of SHOU LAC-1 in PD-related nutritional strategies. However, the evidence remains predominantly associative, and both the causal mechanisms and potential translational relevance require further validation.

## 5. Conclusions

In conclusion, *Lactiplantibacillus plantarum* SHOU LAC-1 administration was associated with partial improvement in motor and defecatory performance in MPTP-treated mice within the preventive intervention paradigm. These changes were accompanied by partial preservation of nigrostriatal TH immunoreactivity, lower α-SYN immunoreactivity, increased expression of neurotrophic-support-related genes, and lower central and colonic inflammatory markers. SHOU LAC-1 administration was also associated with comparatively better-preserved colonic morphology, increased expression of intestinal tight-junction-related genes, higher Nrf2-related antioxidant gene expression, including Nrf2, HO-1, and NQO1, and changes in gut microbial composition and serum metabolic profiles.

Together, these findings indicate coordinated central and peripheral changes following SHOU LAC-1 administration but do not establish causal relationships among gut microbial alterations, systemic metabolism, intestinal changes, and neurological outcomes. These preclinical findings provide preliminary support for further investigation of SHOU LAC-1 as a strain-specific candidate for adjunctive nutritional strategies. Additional mechanistic studies and validation in complementary experimental models are required before its translational relevance to PD can be determined.

## Data Availability

The original contributions presented in this study are included in the article/Supplementary Material. Further inquiries can be directed to the corresponding authors.

## Supplementary Materials

The following supporting information can be downloaded at: https://www.mdpi.com/article/10.3390/foods15173116/s1, Table S1: Primer information used for qRT-PCR; Table S2. Quantitative information for the serum metabolic features shown in Figure 9E,F.
