# Supplementary material for: Preventive Attenuation of MPTP-Induced Parkinson’s Disease-like Abnormalities by Lactiplantibacillus plantarum SHOU LAC-1 in Mice
Source: Foods. 2026 Sep 2;15(17):3116. doi: 10.3390/foods15173116 (PMC13564561; doi:10.3390/foods15173116)
Supplement: Supplementary file 1 [file foods-15-03116-s001.zip › Table S1. Primers information used for qRT-PCR .pdf]

Table S1. Primers information used for qRT-PCR

Primer	Sequence(5'-3')	Location	Amplicon Size （bp）
TH	CCCAAGGGCTTCAGAAGAG	65-83	106
	GGGCATCCTCGATGAGACT	170-152	
TNF-α	CCAGACCCTCACACTCAGA	392-410	187
	GACAAGGTACAACCCATCG	578-560	
IL-1β	CCTGTGTCTTTCCCGTGGA	316-334	357
	GGGTGTGCCGTCTTTCATT	672-654	
IL-6	TTCCATCCAGTTGCCTTCT	275-257	187
	CTCATTTCCACGATTTCCC	392-410	
BDNF	GAAGAGCTGCTGGATGAGGAC	517-537	332
	TTCAGTTGGCCTTTTGATACC	848-828	
Nrf2	GCTGGCTGATACTACCGCTGTT	702-723	167
	CAGTGGAGAGGATGCTGCTGAA	868-847	
HO-1	GCCACCAAGGAGGTACACAT	190-209	103
	AGGAAGCCATCACCAGCTTAAA	292-271	
NQO1	GGTAGCGGCTCCATGTACTCTC	584-605	203
	TCCTCCCAGACGGTTTCCAGAC	786-765	
Gapdh	ACCACAGTCCATGCCATCAC	946-965	452
	TCCACCACCCTGTTGCTGTA	1397-1378	
GDNF	TCACCAGATAAACAAGCGG	297-315	175
	TTTCATAGCCCAAACCCAA	471-453	
IBA-1	TCTGCCGTCCAAACTTGAAGCC	443-464	138
	CTCTTCAGCTCTAGGTGGGTCT	580-559	
GFAP	AAGCAGATGAAGCCACCCTG	3940-3959	625
	GTCTGCACGGGAATGGTGAT	4564-4545	
ZO-1	GGGAGGGTCAAATGAAGACA	6366-6385	145
	GGCATTCCTGCTGGTTACAT	6510-6491	
Occludin	ATAATGGGAGTGAACCCGACG	724-744	187
	CCACGATAATCATGAACCCCA	910-890	
Claudin-1	TGGATGGCTGTCATTGGGG	562-580	127
	ACCTGGCATTGATGGGGGT	688-670	
	ACCTGGCATTGATGGGGGT	688-670	
